# Magnetic and EPR Spectroscopic Studies of Thiolate Bridged Divalent Ni, Pd, and Pt Ions Capped with VO(N_**2**_S_**2**_) Metalloligands

**DOI:** 10.1021/acs.inorgchem.5c05204

**Published:** 2026-01-21

**Authors:** Dakota D. Jones, Manuel Quiroz, Aruzhan Abdikaiym, Akhil K. Singh, Naushad Ahmed, Brad S. Pierce, Marcetta Y. Darensbourg, Kim R. Dunbar

**Affiliations:** † 6865 Department of Chemistry Texas A&M University, College Station, Texas 77843, United States; ‡ Department of Chemistry & Biochemistry, 8059 University of Alabama, Tuscaloosa, Alabama 35487, United States

## Abstract

Reactions of the metallodithiolate complex VO­(bme-dach) (hereafter abbreviated as V, where bme-dach = *N*,*N*′-bis­(2-mercaptoethyl)-1,4-diazacycloheptane) with [Pd^II^(CH_3_CN)_4_]­(BF_4_)_2_ and [Pt^II^(CH_3_CN)_4_]­(BF_4_)_2_ yield the V–*M*–V trimetallic compounds **[VPdV]­(BF**_**4**_)_**2**_ **(2)** and **[VPtV]­(BF**_**4**_)_**2**_ **(3)**. Reaction of a similar metalloligand, VO­(bme-daco) (hereafter abbreviated as V′ where bme-daco = *N*,*N*′-bis­(2-mercaptoethyl)-1,5-diazacyclooctane) with [Ni^II^(CH_3_CN)_6_]­(BF_4_)_2_ afforded the related salt **[V′NiV′]­(BF**_**4**_)_**2**_ **(1)**. X-ray structural analyses revealed that cations in **1**, **2**, and **3** adopt a stairstep C_2h_ structure consisting of two terminal VO­(N_2_S_2_) moieties bridged via thiolate sulfur to the group 10 metal ions. Weak ferromagnetic superexchange coupling (*J* = 0.282 cm^–1^ for **1**, 0.954 cm^–1^ for **2**, and 1.372 cm^–1^ for **3**) was observed between the two *S* = 1/2 V^IV^ centers separated by distances in the range of 5.9–6.3 Å, with *J* values varying following the order Ni < Pd < Pt. Frozen-solution EPR spectra measured on the more soluble [BArF_24_]^−^ (BArF_24_^–^ = tetrakis­((3,5-trifluoromethyl)­phenyl)­borate) analogues revealed that the **[VPtV]^2^** ^**+**^ cation exhibits a 15-line hyperfine splitting of 225 MHz at *g* = 4 in parallel mode, confirming exchange coupling between the two ^51^V, *I* = 7/2 nuclei. Density-functional theory (DFT) calculations indicate an *S* = 1 ground state for **1**–**3**. These results demonstrate that the choice of paramagnetic metallodithiolate ligand and diamagnetic bridge in such trimetallic species influences the sign and magnitude of magnetic interactions.

## Graphical abstract

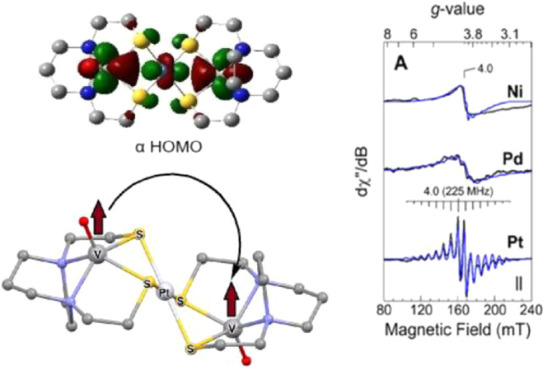

## Introduction

Molecular magnetism research has largely centered around the study of metal spin centers that interact via magnetic superexchange through diamagnetic bridging ligands; such coupling is predicted to be stronger over shorter distances.^1,2^ Ferromagnetic interactions, generated by orthogonality of the metal spin-based magnetic orbitals with the orbitals of the bridging ligand, are less common and generally weaker than antiferromagnetic interactions which require strong overlap between orbitals.^2–6^ An early confirmation of the hypothesis that the magnitude of antiferromagnetic interactions in metal complexes through closed-shell bridging ligands depends on the distance between spin centers is a study published nearly 50 years ago.^7^ These findings notwithstanding, examples of unusually strong magnetic coupling have been reported between metal-based spin centers separated by unusually long distances.^8–10^ Of particular note in this regard is the work of Wieghardt and coworkers who prepared the Cu complex [*L*_2_Cu_2_(OH_2_)_2_(η-terephthalato)]­(ClO_4_)_2_ (*L* = 1,4,7-trimethyl-1,4,7-triazacyclononane) which exhibits unusually strong antiferromagnetic coupling (*J* = −70 cm^–1^) between two *S* = 1/2 Cu^II^ ions, which couple over a distance of 11.25 Å.^8^ More directly related to this work, the trimetallic (Fe–*M*–Fe; *M* = Cr, Fe or Co) complexes, (Figure 1a), bridged by the chelating thiolate ligand, 1,4,7-tris­(4-*tert*-butyl-2-mercaptobenzyl-1,4,7-triazacyclononane) with Fe–Fe distances of 5.7–5.8 Å (Fe–*M* distance 2.9 Å), exhibit antiferromagnetic interactions between the two Fe centers when *M* = Cr or Fe.^11^ The recent report of a vanadium porphyrin dimeric complex, Figure 1b), a dicationic diradical, describes ferromagnetic interactions between two vanadyl centers, V­(IV) d^I^, at a 10 Å separation, yielding a *J* value of 15.3 cm^–1^.^12^

**Figure 1:**
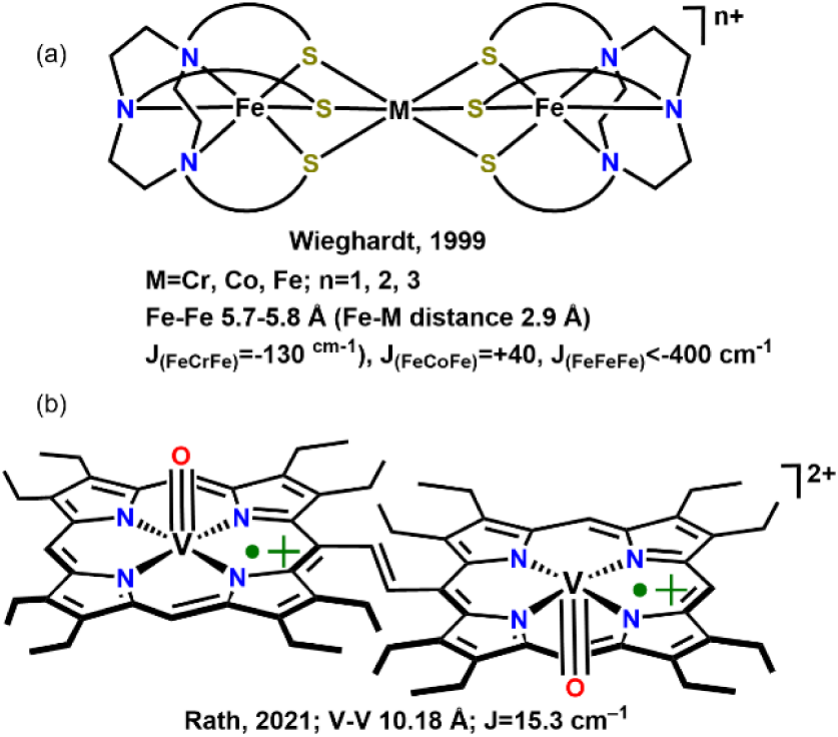
(a) Thiolate bridged Fe–*M*–Fe trimetallics with distances and magnetic coupling constants. (b) An example of ferromagnetic coupling between two vanadyl at >10 Å. Reproduced from ref 11, Copyright 1999 (1a) and ref 12, Copyright 2021 (1b). American Chemical Society.

Thiolate-based bridging ligands are well-known in bioinorganic chemistry where their chemical functions serve as inspiration for synthetic applications toward heterodi- and poly-metallic complexes. For example, tetradentate N_2_S_2_ ligands, inspired by the acetyl Coenzyme A synthase (ACS) enzyme dinickel active site, have been extensively developed as synthons for sulfur-bridged di- and poly-nuclear complexes largely purposed for catalysis;^13^ they are however underdeveloped for applications in the molecular magnetism field. This situation is presumably because of the reputation of instability due to radical-based reactions involving thiolates. This deterrent notwithstanding, there are notable reports of the use of sulfur-based bridges for the design of strongly magnetically coupled compounds.^14^ One example is the dinuclear dysprosium single molecule magnet (SMM), {(C_5_H_4_Me)_2_Dy­(μ-SSiPh_3_)}_2_ in which the use of sulfur bridges in place of the harder oxygen donor atom increased both the magnitude of the antiferromagnetic coupling and the magnetic relaxation barrier.^15^ Related to the research described in our work, several di- and polymetallic complexes derived from metallodithiolates as S-donor ligands provide a convenient backdrop.^16–21^ Of particular note is the use of chelated N_2_S_2_ iron nitrosyl, [Fe­(NO)]^2+^, dithiolate complexes as spin probes in molecular magnetism studies. The preparation and characterization of the compounds [Fe_2_Ni_2_]*X*_2_ (*X* = (PF_6_^–^), (BArF_24_^–^)) (cation shown in Figure 2b) which contains four paramagnetic metal centers is an example of intricate magnetic interaction pathways resulting from a thermodynamically stable coupling of two Ni–S radicals, derived from spin transfer from the (N_2_S_2_)­Fe­(NO) metallodithiolate ligand. This understanding was derived from temperature-dependent magnetic susceptibility studies revealing that the two Fe spins in the {Fe­(NO)}^7^ radical (*S* = 1/2) unit are strongly antiferromagnetically coupled through the central diamagnetic Ni_2_S_2_ bridge at a distance of 8.64 Å, with *J*_4_ = −53.3 cm^–1^.^17^

**Figure 2:**
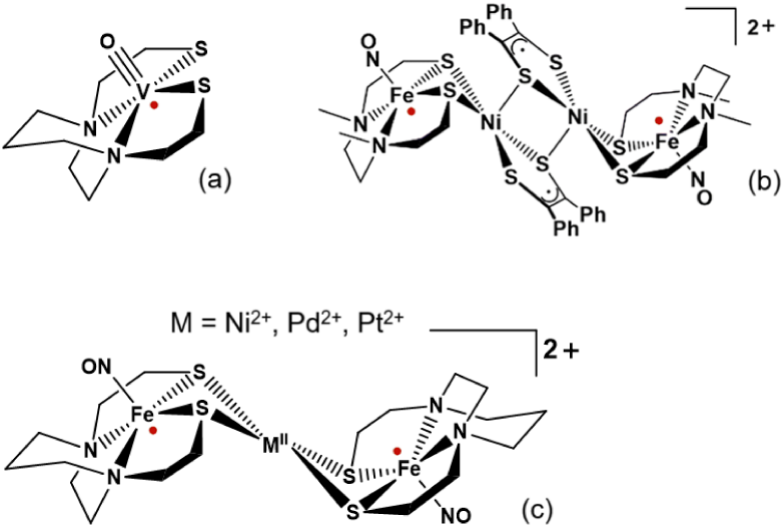
(a) The (VO)­bme-dach ligand. (b) Fe­[Ni_2_S_2_]­Fe^2+^ complex with coupled magnetic centers marked by red dots.^17^ (c) FeMFe complexes with bme-dach ligands and antiferromagnetic coupling.^16^ Structures (b) and (c) reproduced from ref 16 with permission of authors and in accordance with journal policy. Available under a CC-BY NC 3.0 license.

To further investigate this notable finding, analogues of the complex, (Figure 1b) with a single diamagnetic metal atom in place of the Ni_2_S_2_ core were pursued. The group 10 metal ions Ni^II^, Pd^II^ and Pt^II^, were an obvious choice as they adopt square planar geometry in their divalent oxidation state; their valence d-orbitals become increasingly diffuse moving from the first to third row metals. The three cations in [{Fe­(NO)}^7^­(bme-dach)-*M* ^II^-(bme-dach)­{Fe­(NO)}^7^­(BF_4_)_2_ (*M* = Ni, Pd and Pt, bme-dach = *N*,*N*′-bis-mercaptoethyl-1,4-diazacycloheptane, Figure 1c) are similar in their stairstep structures, with Fe–Fe separations in the range of 5.9–6.0 Å. The cations exhibit antiferromagnetic coupling between the two {Fe­(NO)}^7^ units, which increases from Ni (*J* = −3 cm^–1^) to Pd (*J* = −23 cm^–1^) to Pt (*J* = −124 cm^–1^). The consistency of Fe···Fe distances in the series points to a central metal effect that is not based on geometrical or distance differences. Density functional theory calculations revealed that the more diffuse nature of the Pt (5d) orbitals, in comparison to Pd (4d) or Ni (3d) orbitals, enhances the covalent character of the metal–sulfur interaction. The increased mixing between the Pt and S valence orbitals leads to better overlap between the magnetic Fe­(NO) orbitals, accounting for the trend in the observed antiferromagnetic coupling constants.^16^

The intriguing results of the Fe­(NO) thiolate research begged the question of the singularity of the Fe­(NO) paramagnets in this structural motif. Hence, we turned to the vanadyl (V≡O)^2+^ group as another well-known spin probe, with vanadium­(4+) in the d^I^, *S* = 1/2, configuration. Note that we have represented the vanadyl ion as [V≡O]^2+,^ with triple-bond status as described according to the molecular orbital analysis of Ballhausen and Gray.^22^ The vanadyl unit is known to bind within tetradentate N_2_S_2_ ligands, producing a square pyramidal (N_2_S_2_)­(V≡O) neutral complex in which the vanadyl unit is displaced by ca. 0.65 Å above the centroid of the N_2_S_2_ base. We have used both the bme-dach (*N*,*N*′-bis­(2-mercaptoethyl)-1,4-diazacycloheptane) and bme-daco (*N*,*N*′-bis­(2-mercaptoethyl)-1,5-diazacyclooctane) as N_2_S_2_ ligands in this study (Figure 2a).^18,19^

It should be noted that vanadyl compounds are of interest in the field of molecular magnetism for applications in quantum computing. Fundamental design principles for molecular qubits are best studied in simple *S* = 1/2 systems, such as vanadium­(IV) complexes. Contributions from Sessoli^23,24^ and Freedman^25,26^ highlighted how such systems minimize convoluting variables and provide clear insight into qubit behavior. More recently, Zadrozny reported the effect of ligand nuclei on spin relaxation in V­(IV) complexes,^27^ a critical factor since they determine how long information can be stored in a spin’s orientation. Together, these studies position vanadyl compounds as promising candidates for quantum information technologies.

While the Fe­(NO)^2+^ binding center in N_2_S_2_ complexes is also paramagnetic as in the (V≡O)^2+^, an added advantage of the latter is the nuclear signature (^51^V has *I* = 7/2) in electron paramagnetic resonance (EPR) spectroscopy.^19^ In undertaking the current study, we predicted that the magnetic superexchange interaction between the V^IV^ centers would be ferromagnetic. To support this prediction, in {VO­(Hsabhea)}_2_ (Hsabhea = N-salicylidene-2-bis­(2-hydroxyethylamino)­ethylamine) the unpaired electrons in the (V≡O) groups reside in d_*xy*_ orbitals that are orthogonal to the bridge bonds, leading to weak ferromagnetic coupling (*J* = 3.1 cm^–1^).^28^ We hypothesized that, despite the change in sign and magnitude of the magnetic coupling constant, the strength of the magnetic interaction in the group 10 series would follow the same trend as the Fe­(NO) analogues, i.e., increasing in the order Ni < Pd < Pt. Herein we report the syntheses, structures, magnetic properties, EPR spectra, and computational studies of [VO­(bme-daco)-Ni-VO­(bme-daco)]­[BF_4_]_2_ **(1)** and [VO­(bme-dach)-*M*-VO­(bme-dach)]­[BF_4_]_2_ (*M* = Pd **(2)**, Pt **(3)**). Comparisons and contrasts to the previously studied Fe­(NO) analogues will be further elaborated.

## Experimental Section

### Materials

Acetonitrile and diethyl ether were purified by the MBraun Manual Solvent Purification System with Alcoa F200 activated alumina desiccant. Manipulations and reactions were carried out under anaerobic conditions on a Schlenk-line under a N_2_ or Ar atmosphere or in a N_2_ or Ar atmosphere glovebox. Unless otherwise stated, all reagents were used as received from standard vendors such as Sigma-Aldrich, TCI, Ambeed, and BTC. The starting materials *N*,*N*′-bis­(2-mercaptoethyl)-1,4-diazacycloheptane (bme-dach), *N*,*N*′-bis­(2-mercaptoethyl)-1,5-diazacyclooctane (bme-daco), VO­(bme-dach), and VO­(bme-daco) were synthesized according to published procedures. A silica chromatographic column with up to 3% MeOH in CH_2_Cl_2_ was used to isolate VO­(bme-daco).^18,29,30^ The [Ni­(CH_3_CN)_6_]­(BF_4_)_2_ precursor was prepared according to a published procedure.^31^ The [Pt­(CH_3_CN)_4_]­[BF_4_]_2_ precursor was prepared from a modified procedure which involves the protonation of Pt­(acac)_2_ with excess HBF_4_·Et_2_O (64 equiv.) in CH_3_CN. NaBArF_24_ and KBArF_24_ (BArF_24_^–^ = tetrakis­((3,5-trifluoromethyl)­phenyl)­borate) were prepared according to literature procedures.^32,33^ The [*n*-Bu_4_N]­[PF_6_] salt was purchased as reagent grade from Sigma-Aldrich and purified by recrystallizing three times from hot EtOH, followed by vacuum drying at 100 °C for at least 12 h.

### Syntheses

#### [VO­(bme-daco)-Ni^II^-VO­(bme-daco)]­(BF_4_)_2_, [V′–Ni–V′]­(BF_4_)_2_ **(1)**

VO­(bme-daco) (32.6 mg, 0.109 mmol) was suspended in 5 mL of CH_3_CN and [Ni^II^(CH_3_CN)_6_]­(BF_4_)_2_ (27 mg, 0.051 mmol) was added as a powder. The mixture was stirred at room temperature for 16 h under Ar and the resulting dark purple solution was filtered through a small Celite plug to remove unreacted VO­(bme-daco). Dark purple crystals of **1·CH**_**3**_ **CN** (yield: 33.2 mg, 74%) were grown by vapor diffusion of diethyl ether into the filtered CH_3_CN solution at room temperature. UV–vis absorption spectrum [CH_3_CN, λ_max_, nm (ε_M_, M^–1^ cm^–1^)]: 299 (12080), 402 (706), 503 (268). ATR-IR on solid: ν­(V≡O) = 992 cm^–1^. ESI-MS positive mode: [(VO)_2_NiC_20_H_40_N_4_S_4_]^2+^ = 328.01 *m*/*z*.

#### [VO­(bme-dach)-Pd^II^-VO­(bme-dach)]­(BF_4_)_2_, [V–Pd–V]­(BF_4_)_2_ **(2)**

In separate vials, VO­(bme-dach) (63 mg, 0.14 mmol) was suspended in 7 mL of CH_3_CN and [Pd^II^(CH_3_CN)_4_]­(BF_4_)_2_ (44 mg, 0.098 mmol) was dissolved in 3 mL of CH_3_CN. The Pd^II^(CH_3_CN)_4_(BF_4_)_2_ solution was added dropwise to the VO­(bme-dach) suspension, and the mixture was stirred at room temperature for 18 h under N_2_. The resulting olive-green solution was filtered through a small Celite plug to remove excess VO­(bme-dach). Green X-ray quality crystals of **2·2CH**_**3**_ **CN** (yield: 53 mg, 64%) were grown by vapor diffusion of diethyl ether into the filtered CH_3_CN solution at room temperature. UV–vis absorption spectrum [CH_3_CN, λ_max_, nm (ε_M_, M^–1^ cm^–1^)]: 227 (20480), 255 (21080), 317 (23000), 613 (137). ATR-IR on solid: ν­(V≡O) = 998 cm^–1^. ESI-MS positive mode: [(VO)_2_PdC_18_H_36_N_4_S_4_]^2+^ = 337.98 *m*/*z*. Anal. Calcd for C_18_H_36_N_4_O_2_S_4_V_2_B_2_F_8_Pd + 1 CH_3_CN (F.W. 891.73): C, 26.94; H, 4.41; N, 7.85. Found: C, 27.17; H, 4.56; N, 8.09.

#### [VO­(bme-dach)-Pt^II^-VO­(bme-dach)]­(BF_4_)_2_, [V–Pt–V]­(BF_4_)_2_ **(3)**

In separate vials, VO­(bme-dach) (40 mg, 0.14 mmol) was suspended in 4 mL of CH_3_CN and [Pt^II^(CH_3_CN)_4_]­(BF_4_)_2_ (27 mg, 0.051 mmol) was dissolved in 2 mL of CH_3_CN. The Pt^II^(CH_3_CN)_4_(BF_4_)_2_ solution was added dropwise to the VO­(bme-dach) suspension, and the mixture was stirred at room temperature for 24 h under N_2_. The resulting brown solution was filtered through a small Celite plug to remove excess VO­(bme-dach). Green-brown X-ray quality crystals of **3·2CH**_**3**_ **CN** (yield: 35 mg, 72%) were grown by vapor diffusion of diethyl ether into the filtered CH_3_CN solution at room temperature. UV–vis absorption spectrum [CH_3_CN, λ_max_, nm (ε_M_, M^–1^ cm^–1^)]: 241 (25200), 265 (28200), 377 (754), 583 (100). ATR-IR on solid: ν­(V≡O) = 998 cm^–1^. ESI-MS positive mode: [(VO)_2_PtC_18_H_36_N_4_S_4_]^2+^ = 382.51 *m*/*z*. Anal. Calcd for C_18_H_36_N_4_O_2_S_4_V_2_B_2_F_8_Pt (F.W. 939.94): C, 23.02; H, 3.86; N, 5.96. Found: C, 22.72; H, 3.95; N, 5.85.

The more soluble [BArF_24_]^−^ analogues (BArF_24_^–^ = tetrakis­((3,5-trifluoromethyl)­phenyl)­borate) [VO­(bme-daco)-Ni^II^-VO­(bme-daco)]­(BArF_24_)_2_, [V′–Ni–V′]­(BArF_24_)_2_. **(4)**, [VO­(bme-dach)-Pd^II^-VO­(bme-dach)]­(BArF_24_)_2_, [V–Pd–V]­(BArF_24_)_2_, **(5)**, and [VO­(bme-dach)-Pt^II^-VO­(bme-dach)]­(BArF_24_)_2_, [V–Pt–V]­(BArF_24_)_2_. **(6)** were prepared and used for the EPR and electrochemical studies. The syntheses are provided in Scheme S1.

### Physical Measurements

Mass spectrometry (ESI-MS) measurements (Figures S1–S3) were performed at the Chemistry Mass Spectrometry Facility at Texas A&M University. Electronic absorption spectra (Figure S4) were obtained using a Shimadzu UV-1601PC spectrophotometer. IR spectra were recorded on an ATI Mattson Genesis Series FTIR spectrometer with ATR-IR attachment. EPR spectra were recorded using a Bruker ELEXSYS E540 X-band spectrometer with a ColdEdge Stinger closed-loop liquid helium cryosystem inserted into an Oxford ESR900 cryostat. A LakeShore 336 temperature controller was used to regulate sample temperature. Elemental analyses were performed at the Atlantic Microlab Inc., located in Norcross, GA.

### Electron Paramagnetic Resonance Spectroscopy

Samples used for EPR studies were prepared at a concentration of 10 mM in dry acetonitrile under strict anaerobic conditions, frozen in liquid nitrogen, and then shipped to the University of Alabama for EPR studies. Continuous wave (CW) X-band EPR experiments were performed at the University of Alabama EPR facility using a Bruker ELEXSYS E540 X-band spectrometer (Bruker-Biospin Billerica, MA). Cryogenic measurements were made using a ColdEdge Stinger closed-loop liquid helium cryosystem inserted into an Oxford ESR900 cryostat. A LakeShore 336 temperature controller was used to regulate sample temperature. EPR simulations were calculated using SpinCount (ver. 8.0.9019.20391) developed by Hendrich at Carnegie Mellon University by utilizing the general spin Hamiltonian shown in eq 1.^34–36^ For a pair of spin-coupled vanadyl ions with electronic spins (*S*_1_ = *S*_2_ = 1/2) and nuclear spins (*I*_1_ = *I*_2_ = 7/2), the spin-Hamiltonian can be expressed as follows:

1 $$Ĥ=-2JS_{1}S_{2}+{Ĥ}_{dip}+{Ĥ}_{1}+{Ĥ}_{2}$$

In the above expression, ***J*** represents the isotropic Heisenberg–Dirac–van Vleck electronic exchange constant between each ^51^V-site. Dipolar interactions are introduced by *Ĥ*_dip_, and *Ĥ_i_* (i = 1, 2) are the intrinsic spin Hamiltonians corresponding to each vanadyl ion. The dipolar spin–spin interactions were calculated using eq 2,

2 $${Ĥ}_{dip}=\frac{\mu_{0}}{4\pi }\beta^{2}\left[\frac{\left(S_{1}\cdot g_{1}\right)\cdot \left(S_{2}\cdot g_{2}\right)}{r^{3}}-\frac{3\cdot \left(S_{1}\cdot g_{1}\cdot r\right)\cdot \left(S_{2}\cdot g_{2}\cdot r\right)}{r^{5}}\right]$$

where ***r*** represents the internuclear vector and ***g***_***1***_ and ***g***_***2***_ are the intrinsic *g*-tensors for individual vanadyl ions. The dipolar interaction is calculated using the distance separating the two ^51^V-sites (*r*) and the dipolar angles (θ, ϕ) relating the coordinate system for each V-sites. eq 3 presents the spin Hamiltonian for individual vanadyl ions *Ĥ_i_* (i = 1,2). As vanadyl ions have an electronic spin (*S*_1_ = *S*_2_ = 1/2), there are no zero field splitting (SDS) contributions to the Hamiltonian. Consequently, the energy for each site simplifies down to the sum of electronic Zeeman and nuclear hyperfine terms.

3 $${Ĥ}_{i}=\beta S_{i}\cdot {\mathrm{g̃}}_{i}\cdot B+S_{i}\cdot {Ã}_{i}\cdot I_{i}\;(i=1,2)$$

Here, ***g̃*_*i*_** and ***Ã***_***i***_ represent the intrinsic ***g*** and ***A***-tensors for individual ^51^V-sites. Hyperfine coupling from the two ^51^V-sites was treated by second-order perturbation theory.^37,38^ Simulations were generated with consideration of all intensity factors, both theoretical and experimental, to allow for determination of species concentration. The only unknown factor relating the spin concentration to signal intensity was an instrumental factor that is specific to the microwave detection system. This factor was determined by a 1.0 mM Cu­(EDTA) spin standard prepared from a copper atomic absorption standard solution purchased from Sigma-Aldrich. All samples were prepared in custom-made 3 mm Quartz EPR tubes made at the University of Alabama glassblowing facility. All spectra were collected under nonsaturating conditions at a modulation frequency of 100 kHz.

### X-ray Crystallography

The structure of [VNiV′]­(BF_4_)_2_·CH_3_CN (**1·CH**_**3**_ **CN)** was measured on a Rigaku XtaLAB Synergy-S diffractometer at 100 K with a Cu X-ray tube (*K*_α_ = 1.5418 Å). The crystal structures of [VPdV]­(BF_4_)_2_·2CH_3_CN (**2·2CH**_**3**_ **CN)** and [VPtV]­(BF_4_)_2_·2CH_3_CN (**3·2CH**_**3**_ **CN)** were measured using a BRUKER Quest X-ray (fixed-Chi geometry) diffractometer at 110 K with a Mo–Iμs X-ray tube (*K*_α_ = 0.71073 Å). Integrated intensity information for each reflection was obtained by reduction of the data frames with the program APEX4.^39^ The data were merged and scaled to produce a suitable dataset. The absorption correction program SADABS was employed to correct data for absorption effects.^40^ Solutions were obtained readily using Olex2^41^, with ShelXT^42^ being used to solve the structure. The structure of **1·CH**_**3**_ **CN** was solved and refined in the *C*2/*c* space group with *Z* = 4 and *Z*′ = 0.5, and **2·2CH**_**3**_ **CN** and **3·2CH**_**3**_ **CN** were solved and refined in the *P*2_1_/*c* space group with *Z* = 2, and *Z*′ = 0.5. Hydrogen atoms were placed in idealized positions and were set to ride on the respective parent atoms. All non-hydrogen atoms were refined with anisotropic thermal parameters. Absence of additional symmetry or voids were confirmed using PLATON (ADDSYM).^43^ The structures were refined (ShelXL, weighted least-squares refinement on *F* ^2^) to convergence using Olex2.^41^ Powder X-ray diffraction patterns were collected on a BRUKER D8 Endeavor diffractometer with a 1 kW Cu X-ray tube, operating at a potential of 40 kV and a current of 25 mA.

### Electrochemistry

Cyclic voltammetry experiments were performed at room temperature under an N_2_ atmosphere on a Pine WaveNow^XV^ 2620303 electrochemical workstation with acetonitrile as solvent and 0.1 M tetra-*n*-butylammonium hexafluorophosphate (N-*n*Bu_4_PF_6_) as the electrolyte. The setup used a glassy carbon working electrode, a platinum wire counter electrode, and a Ag/Ag^+^ reference electrode. The concentration of the [V*M*V]­(BArF_24_)_2_ complexes used for cyclic voltammetry was 1 mM. At the end of the experiments, ferrocene was added as an internal standard.

### Magnetic Measurements

DC magnetic measurements were performed on freshly prepared, crushed crystalline samples that were tightly folded in a plastic bag and packed into a straw. Data were measured from 2 to 300 K in applied magnetic fields of 0.1 and 1 T on a Quantum Design SQUID, Model MPMS with a 7 T magnet. The diamagnetic contributions from the plastic bag and the straw were subtracted from the raw data and Pascal’s constants were used to subtract the diamagnetic corrections of the atoms from the experimental susceptibilities.^44^ Temperature-independent paramagnetism (TIP) corrections were applied based on the best-fit values supplied by the PHI software. Magnetic data were fitted using the PHI software program.^45^

### Computational Methods

Density functional theory (DFT) calculations were performed in Gaussian 16 Revision C.01 ^46^ with the TPSSTPSS^47^ functional. The triple-ζ basis set 6–311++G­(d,p) was used for hydrogen, carbon, sulfur, nitrogen, and oxygen atoms^48–50^ while the Wachters–Hay basis set, 6–311++G­(d,p),^51–53^ was used for vanadium and nickel atoms. For palladium and platinum atoms, an Effective Core Potential (ECP) and a triple-ζ quality basis set (cc-pVTZ-PP) was used for core and valence electrons, respectively.^54,55^ Crystal structures of **1·CH**_**3**_ **CN**, **2·2CH**_**3**_ **CN**, and **3·2CH**_**3**_ **CN** were imported to use as the starting coordinates for gas phase optimization and frequency calculations using GaussView 6.1.1.^56^ Likewise, the initial geometries of the cations for optimization in the open-shell singlet state was based on the optimal geometry of the triplet compounds. All species were confirmed to be minimum energy structures by the absence of imaginary frequencies.

## Results and Discussion

Compounds **[VPdV]­(BF**_**4**_)_**2**_ **(2)** and **[VPtV]­(BF**_**4**_)_**2**_ **(3)** were obtained as described in the Experimental section (Scheme 1). Attempts to prepare the analogous **V–Ni–V** compound gave promising results according to initial MS data on the reaction solution, but XRD on red crystals isolated from the mother liquor indicated that a Ni atom had displaced the V≡O groups from the N_2_S_2_ pockets, an exchange that is attributed to the preference of the nickel over vanadyl for the N_2_S_2_ coordination site.^18,21^ The mass spectrum showed a signal around *m*/*z* = 306 which supports the fact that Ni displaces the (V≡O) unit from the N_2_S_2_ coordination site in bme-dach. Use of the different VO­(N_2_S_2_) metalloligand, VO­(bme-daco), however, led to the isolation of the similar trinuclear complex, **[V′NiV′]­(BF**_**4**_)_**2**_ **(1)** (bme-daco = bis-mercaptoethyl-1,5-diazacyclooctane).

**Scheme 1:**
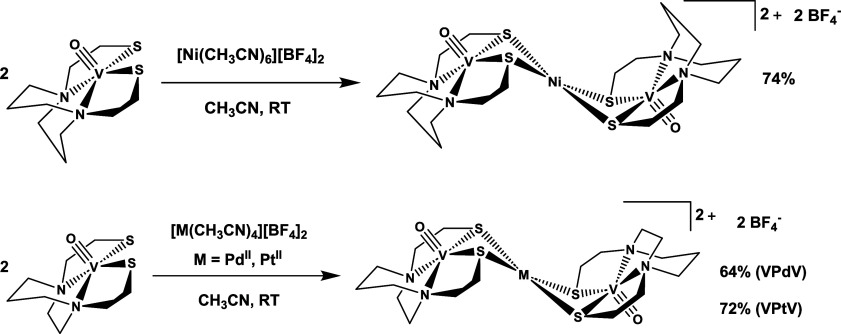
Preparation of **[V′NiV′]­(BF**_**4**_)_**2**_ **(1), [VPdV]­(BF**_**4**_)_**2**_ (**2**), and **[VPtV]­(BF**_**4**_)_**2**_ **(3)** with Percent Yields

### X-ray Crystallography

The structures of **1·CH**_**3**_ **CN, 2·2CH**_**3**_ **CN** and **3·2CH**_**3**_ **CN** (Figure 3; see Figures S21–S23 for packing diagrams, Table S2 for crystal and refinement parameters, and Tables S5–S10 for selected bond lengths and angles) revealed that the [VON_2_S_2_]^2+^ cations adopt a structure akin to the previously synthesized [Fe*M*Fe]^2+^ analogues. As in the case of the Fe compounds, the cations exhibit C_2h_ symmetry which leads to identical bond distances and displacements on either side of the central Pd or Pt atom. The V atom displacement out of the N_2_S_2_ plane, however, is significantly greater (0.792 Å for **2** and 0.790 Å for **3**) than the Fe atom displacement in the related [FePdFe]^2+^ and [FePtFe]^2+^ cations (0.589 Å).^13^ In the case of **1**, the V atom displacement out of the N_2_S_2_ plane (0.694 Å) is also greater than that of [FeNiFe]^2+^ (0.615 Å) but is less pronounced, indicating a reduced effect of the vanadyl unit on the bridging sulfurs. The distance between the two V atoms (6.227 Å for **2** and 6.228 Å for **3**) is also comparable to the Fe–Fe distance in [FePdFe]^2+^ (6.017 Å) and [FePtFe]^2+^ (5.941 Å). The V–S distance and S–V–S bite angles also are smaller for **1** than for **2** or **3**. Hinge angles (Table 1; defined as the angle between the N_2_S_2_ and *M*S_4_ planes) are also similar, but slightly more acute than those in the FeNO analogues (104.8° for [FePdFe]^2+^, 102.3° for **[VPdV]** ^**2**+^, 102.3° for [FePtFe]^2+^, 100.6° for **[VPtV]** ^**2+**^, 106.4° for [FeNiFe]^2+^ and 97.9° for **[V′NiV′]** ^**2+**^), leading to a more obvious stair-step shape. The V–O distance is slightly contracted (1.588 Å for **2**, and 1.594 Å for **3**) compared to 1.605 Å in the free ligand which is ascribed to the donation of electron density to the central metal ion, reducing its donor effect on the vanadyl unit. The S–S separation is also shorter, 3.175 or 3.183 Å compared to 3.542 Å. For **1**, the V–O distance (1.592 Å) is slightly less than in the free ligand (1.600 Å). In all three compounds, the V displacement from the N_2_S_2_ plane is protracted; it is only 0.652 Å in the free ligand. To understand the extent of geometry distortion from the perfect square pyramidal geometry, we performed continuous shape measurement (CShM) analysis^57^ on each of the V^IV^ centers in **1**–**3** (Table 2). The CShM value of zero represents perfect geometry, while a deviation from zero indicates the extent of deviation from the perfect shape. Among all the possible geometries for five-coordinate V^IV^ ions, the smallest deviation of the CShM values was found for the square pyramidal geometry (see the bold values in Table 2). We also observed that the distortion from the square pyramidal geometry around the V^IV^ ions increases with the replacement of Ni^II^ by Pd^II^ or Pt^II^.

**Figure 3:**
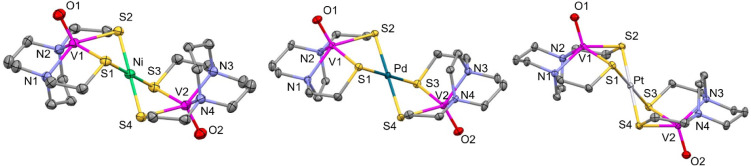
Structures of the dications in Left: [VO­(bme-daco)-Ni-VO­(bme-daco)]­(BF_4_)_2_·CH_3_CN (**1·CH**_**3**_ **CN**), Center: [VO­(bme-dach)-Pd-VO­(bme-dach)]­(BF_4_)_2_·2CH_3_CN **(2·2CH**_**3**_ **CN**), and Right: [VO­(bme-dach)-Pt-VO­(bme-dach)]­(BF_4_)_2_·2CH_3_CN (**3·2CH**_**3**_ **CN**).

**Table 1:** Key Metrical Parameters for the [V ***M*** V]^**2+**^ Dications^a^

	[V′NiV′]^2+^		[VPdV]^2+^		[VPtV]^2+^	
Parameter	**Exp.**	**Calc.**	**Exp.**	**Calc.**	**Exp.**	**Calc.**
V–V distance (Å)	5.979(9)	6.197	6.272(11)	6.493	6.228(12)	6.430
V–*M* distance (Å)	2.990(4)	3.099	3.136(5)	3.246	3.114(6)	3.215
Average V–S distance (Å)	2.352(7)	2.380	2.373(8)	2.400	2.380(7)	2.404
Average *M*–S distance (Å)^b^	2.228(6)	2.256	2.334(6)	2.376	2.335(6)	2.373
S–V–S Bite Angle (°)	79.8	80.5	84.0	84.7	84.0	84.6
Hinge (°)^1^	97.9°	104.5°	102.3°	108.5°	100.6°	106.0°

a Interplanar angle between the *M*S_4_ plane and the N_2_S_2_ best-fit plane; see Figure S20.

**Table 2:** Continuous Shape Measurement on V^IV^ Sites in Complexes **1–3**

	VNiV **(1)**		VPdV **(2)**		VPtV **(3)**	
	**V1**	**V2**	**V1**	**V2**	**V1**	**V2**
PP-5	30.394	30.394	31.542	31.542	31.442	31.442
vOC-5	1.965	1.965	2.792	2.792	2.783	2.783
TBPY-5	6.338	6.338	6.437	6.437	6.448	6.448
SPY-5	**0.961**	**0.961**	**1.107**	**1.107**	**1.119**	**1.119**
JTBPY-5	8.068	8.068	8.526	8.526	8.520	8.520

PP-5: Pentagon (D_5h_), vOC-5: Vacant octahedron (C_4v_), TBPY-5: Trigonal bipyramid (D_3h_), SPY-5: Spherical square pyramid (C_4v_), JTBPY-5: Johnson trigonal bipyramid J12 (D_3h_)

Bulk samples of **1·CH**_**3**_ **CN, 2·2CH**_**3**_ **CN** and **3·2CH**_**3**_ **CN** (70–120 mg) were ground to a fine powder for powder X-ray diffraction analysis (Figure S24). Powder diffraction peaks at lower 2θ angles were observed at similar positions to those predicted from simulations on the single-crystal diffraction data, but the less intense peaks at higher 2θ angles showed less precise overlap. This difference is attributed to loss of crystallinity and solvent of crystallization during sample preparation, which may be expected as all three compounds contain interstitial acetonitrile molecules in their crystal structures.

Since the [V*M*V]­(BF_4_)_2_ salts are only sparingly soluble in acetonitrile, which rendered them unsuitable for solution EPR spectral studies and cyclic voltammetry measurements, metathesis with NaBArF_24_ or KBArF_24_ was performed to afford crystals of **[V′NiV′]­(BArF**_**24**_)_**2**_ **·2CH**_**2**_ **Cl**_**2**_ (**4·2CH**_**2**_ **Cl**_**2**_) and **[V*M*V]­(BArF**_**24**_)_**2**_ **·2CH**_**2**_ **Cl**_**2**_ (*M* = Pd, **5·2CH**_**2**_ **Cl**_**2**_, *M* = Pt, **6·2CH**_**2**_ **Cl**_**2**_) (see Scheme S1 and Table S3). The [V ***M*** V]^**2+**^ units observed in the X-ray structures of these salts are identical to those found in the [BF_4_]^−^ salts, although two equivalents of CH_2_Cl_2_ cocrystallize with the product in these compounds. In the cases of **4** and **6**, disordered hexanes (**4**) and dichloromethane (**6**) and were observed in the interstices of the crystals. For **4**, the disordered hexane was accounted for by including a solvent mask with 0.68 molecules, accounting for 34 electrons in solvent voids. For **6**, 1.805 molecules of dichloromethane were modeled using appropriate constraints and restraints while an additional 0.615 molecules were included in a solvent mask calculated by OLEX2, accounting for 103 electrons in the solvent voids.

### Magnetic Measurements

Crystals of **1·CH**_**3**_ **CN**, **2·2CH**_**3**_ **CN** and **3·2CH**_**3**_ **CN** were crushed to a fine powder for direct current (dc) magnetic susceptibility measurements, which were performed from 2 to 300 K under applied fields of 0.1 T (Figure S16) and 1 T (Figure 4). Field-dependent magnetization measurements performed from 0 to 7 T at temperatures of 2–5 K indicate that 1 T is still in the linear region for all three compounds (Figure S18). For all three compounds, the room-temperature χ_m_ *T* value is between 0.67 and 0.69 emu mol/K, suggesting a *g* factor less than 2 (for *g* = 2 the χ_m_ *T* is 0.75 emu mol/K for two noninteracting *S* = 1/2 ions). The χ_m_ *T* data for all three compounds exhibit an upward slope at higher temperatures, requiring a TIP contribution (0.284 × 10^–3^ emu mol/K for **1**, 0.053 × 10^–3^ emu mol/K for **2**, and 0.897 × 10^–3^ emu mol/K for **3**). The χ_m_ *T* values increase below ∼15 K, reaching a maximum of 0.73 emu mol/K for **1**, 0.83 emu mol/K for **2** and 0.87 emu mol/K for **3** (See Figure 4; χ_m_ vs *T* plots are provided in Figure S17). The slight increase of χ_m_ *T* at low temperature indicates the presence of weak ferromagnetic coupling between the two V^IV^ spin centers, consistent with the work of Plass^21^ and the predicted properties from DFT calculations (see computational section). The low temperature 2–100 K 1/χ_m_ data were fitted to the Curie–Weiss law for all the complexes **1**–**3** (Figure S15). The obtained θ values of 0.41, 0.49, and 1.06, for complexes **1**, **2**, and **3** respectively are in accord with magnetic exchange increasing in the order **[V′NiV′]** ^**2+**^ < **[VPdV]** ^**2+**^ < **[VPtV]** ^**2+**^.

**Figure 4:**
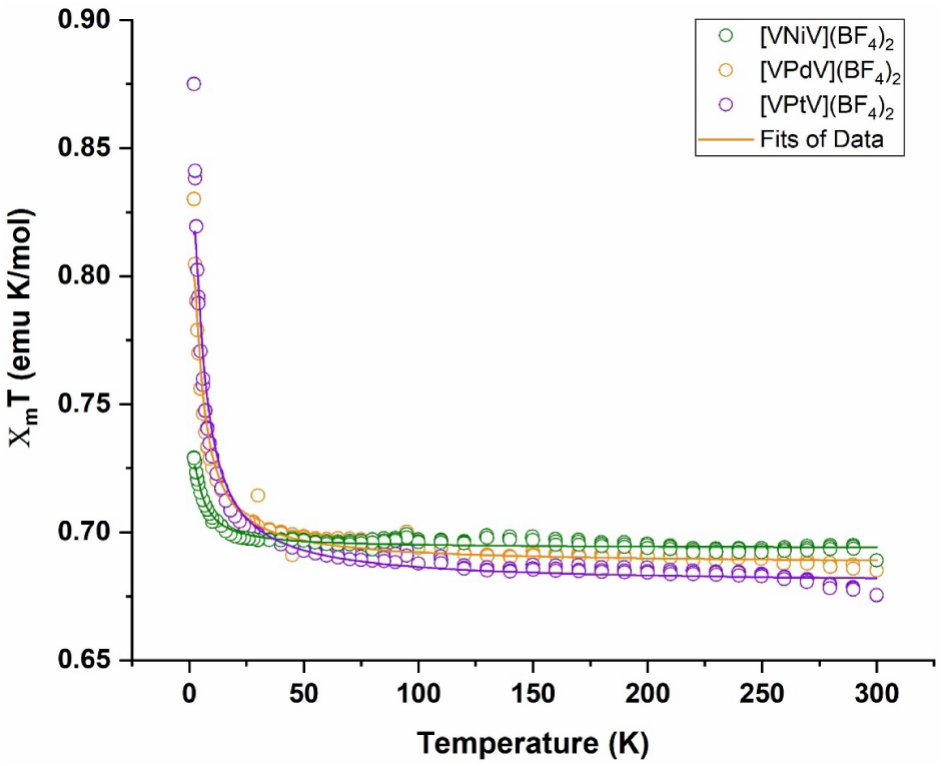
χ_m_ *T* vs *T* plots, at 1 T, for **1** (green circles), **2** (orange circles) and **3** (purple circles). Solid lines are the fits given by PHI. Fit parameters: **1**: *g* = 1.92, *J* = 0.282 cm^–1^, TIP = 0.284 * 10^–3^ emu*mol. **2**: *g* = 1.91, *J* = 0.954 cm^–1^, TIP = 0.053 × 10^–3^ emu*mol, **3**: *g* = 1.90, *J* = 1.372 cm^–1^, TIP = 0.897 × 10^–3^ emu*mol.

To estimate the magnitude of the magnetic coupling constant *J*, PHI software was used with the following Hamiltonian (eq 4)­

4 $$H=\mu_{B}H\left(gS_{1}+gS_{2}\right)-2J\left(S_{1}\cdot S_{2}\right)$$

where the first term accounts for Zeeman splitting (μ_B_ *is the Bohr magneton* and *H* is the field) and the *S*_1_ and *S*_2_ in the second term accounts for the *S* = 1/2 spins on the two vanadyl ions. The magnetic susceptibility data were fitted for the complexes, using parameters *g* and *J*, which were allowed to vary freely. The best fits were obtained using *g* = 1.92 and *J* = 0.282 cm^–1^ for **1,** *g* = 1.91 and *J* = 0.954 cm^–1^ for **2**, and *g* = 1.90 and *J* = 1.372 cm^–1^ for **3**. To better understand the reliability of fitted parameters, magnetic data fitting was also carried out with either *g* or *J* fixed one at a time at values ±0.01 (greater and less than those obtained above). A slight change in the resulting *g* or *J* parameters (Table S1 in ESI) was observed, but *J* remains positive for all the cases, indicating that the sign and magnitude of fitted *J* values are reliable.

While all three complexes exhibit ferromagnetic coupling between V^IV^ ions over a distance of ∼6 Å, the magnitude of the coupling is much weaker than the antiferromagnetic interactions observed for the FeNO analogues ([FeNiFe]­(BF_4_)_2_: −3 cm^–1^; [FePdFe]­(BF_4_)_2_; −23 cm^–1^; and [FePtFe]­(BF_4_)_2_: −124 cm^–1^).^1^ This result is not unexpected, given the greater displacement of the V atom from the N_2_S_2_ plane as compared to the Fe atoms in the Fe­(NO) analogues, which limits interaction between the magnetic V d_*xy*_ orbital and the sulfur p orbitals. Also, importantly, the d_*xy*_ magnetic orbital is not involved in sulfur bonding to the metal atoms which renders magnetic interactions weaker. As the central metal is varied from Ni to Pd to Pt, the magnitude of the coupling constant between the V^IV^ spins increases, following the same trend observed in the FeNO analogues, but opposite in sign (see Figure S14 for comparison). When the magnetic field strength was reduced to 0.1 T, the magnetic coupling strengths and required TIP corrections were somewhat different (*J* = 0.258 cm^–1^ for **1**, 1.064 cm^–1^ for **2** and 1.374 cm^–1^ for **3**) but the *M* = Ni < Pd < Pt trend remains unchanged (Figure S16). Additionally, PHI fits were carried out with *g* and *J* fixed at values 0.01 greater and less than those found when both parameters were allowed to vary freely. When the *g*-factor was changed, the value of *J* changed by only ∼0.1–0.2 cm^–1^ (Table S1), indicating that the fitted *J* values were reliable. These data provide additional evidence that the more diffuse 5d orbitals are indeed better suited to interact with the sulfur p orbitals and to transfer spin polarization between the two magnetic metal centers.

### Electrochemistry

Cyclic voltammograms of solutions of crystalline **[V′NiV′]­(BArF**_**24**_)_**2**_ **·2CH**_**2**_ **Cl**_**2**_ (**4·2CH**_**2**_ **Cl**_**2**_) and **[V*M*V]­(BArF**_**24**_)_**2**_ **·2CH**_**2**_ **Cl**_**2**_ (*M* = Pd, **5·2CH**_**2**_ **Cl**_**2**_, *M* = Pt, **6·2CH**_**2**_ **Cl**_**2**_) (Figures S6–S8) were obtained in dry acetonitrile (stored over molecular sieves) in a plastic purge box under a dinitrogen atmosphere. All E values are referenced to ferrocene (Fc^+^/Fc) added as an internal standard at the end of the experiment. Unlike the [Fe**–*M*–**Fe] complexes, the three members of the [V**–*M*–**V] triad exhibit complex redox properties with no reversible events. All three compounds exhibit broad reduction features between −1 and −2 V, which is attributed to reduction of the coordinated VO^2+^ unit in accord with the electron-withdrawing character of the *M*S_4_ bridge which renders the vanadyl reduction more favorable. Both **4** and **5** exhibit an irreversible feature at potentials (−2.54 V for **4** and −2.60 V for **5**, referenced to Fc^+^/Fc; see Figures S5–S6 and S9–S10) similar to the VO^2+^→ VO^+^ reduction event observed in the cyclic voltammograms of the VO­(bme-daco) and VO­(bme-dach) metalloligands (−2.59 and −2.54 V respectively).^19,58^ We interpret these results to indicate lability of the Ni^II^ and Pd^II^ ions which releases the monomers VO­(bme-daco) and VO­(bme-dach). There was no observable VO^2+^ to VO^+^ reduction peak^19^ indicative of the monomer in the case of **6** which we attribute to a stronger Pt^II^ interaction with the sulfur atoms (Figure S7; see Figure S13 for the [Pt^II^(MeCN)_4_]^2+^ precursor). The VO­(bme-dach) and VO­(bme-daco) precursors exhibit thiolate-based oxidations at +0.3 V, and this VO­(bme-dach) redox event was clearly observed in the cyclic voltammogram of **4**. While **5** and **6** also display oxidation events in this range, they appear only on return sweeps after the voltage has first reached sufficiently negative potentials; the presence of heavy group 10 metals apparently suppresses sulfur oxidation. In addition to the sulfur-based oxidation at +0.35 V, compound **4** exhibits an additional oxidation at +1.15 V, which may be another S-based event due to the presence of the different bme-daco ligand or potentially a V^IV^ to V^V^ oxidation.^58^

Finally, **5** displays a unique reduction event at −0.95 V that is not observed in the other two compounds. While it is irreversible at lower scan rates (<0.4 V/s) at faster rates, above 2 V/s, this reduction becomes quasi-reversible with *E*_pc_ – *E*_pa_ = 99 mV (Figure S8). This reduction is only observed for **[VPdV]­(BArF**_**24**_)_**2**_. Irreversibility of the reduction suggests that the V–Pd^I^–V intermediate is chemically unstable. Additionally, when the potential is swept back to the positive direction, an oxidation at +0.07 V appears after the compound has undergone this reduction. This may be due to reoxidation of Pd^I^ to Pd^II^ as evidenced by the similar Pd^I^ → Pd^II^ reduction observed for the [Pd^II^(MeCN)_4_]­{BF_4_}_2_ precursor (Figure S12). We postulate that the acetonitrile solvated cation, [Ni^II^(MeCN)_6_]^2+^ is released from **4** after passing through the reduction at −2.54 V; the Ni^I^ to Ni^II^ reoxidation observed in the [Ni^II^(MeCN)_4_]­{BF_4_}_2_ precursor (Figure S11) overlaps with the thiolate S oxidation in VO­(bme-daco), thus only one peak would be expected, as is observed (Figure S5).

### Electron Paramagnetic Resonance Spectroscopy

Vanadyl synthons are easily characterized using EPR spectroscopy; by far the most abundant isotope is ^51^V, spin 7/2, which avoids complication of the spectra by isotopes of different nuclear spins. The one d electron spin, when coupled to the nuclear spin, gives a characteristic eight-line hyperfine splitting pattern. For vanadyl (V≡O) compounds, EPR spectra are generally axial due to the strong V–O bond. Vanadyl incorporation into the active sites of enzymes, including pyruvate carboxylase and chloroplast F1-ATPase, has been used to identify the amino acids involved in binding to the metal cofactor and the effects of changing specific residues.^59^ When two vanadyl groups couple to each other with a triplet ground state, a 15-line EPR spectrum is observed, although the pattern may be broadened depending on zero-field splitting.^60^

Samples of **[V′–Ni** ^**II**^ **–V′]­(BArF**_**24**_)_**2**_ **(4)** and [V ***M*** V**]­(**BArF_**24**_)_**2**_ [*M* = Pd^II^ (**5**), Pt^II^ (**6**)] were characterized by CW X-band EPR with microwave field polarization both parallel (A) and transverse (B) to the static magnetic field (Figure 5). For this series, signal intensities were scaled to simplify comparison. All samples exhibit a resonance in parallel mode (Figure 5, panel A) at *g* ∼ 4.0. As shown in Figure 6 (right), this transition is assigned to a Δ*m*_s_ > ±1 transition (levels 1→3) within the *S* = 1 spin manifold. For all complexes, the temperature dependence of the *g* ∼ 4 signal deviates from Curie-law in that the temperature-normalized signal intensity (*S* × *T*) decreases with increasing temperature. This observation is consistent with depopulation of a ground state (*S* = 1) spin-manifold and ferromagnetic coupling between ^51^V-sites. Data from SQUID magnetometry indicate a coupling constant *J* of <2 cm^–1^ for all three complexes with room temperature behavior consistent with two noninteracting *S* = 1/2 centers. Predictions from DFT calculations suggest a value of *J* less than 1 cm^–1^ for these complexes (vide infra), so it should not be surprising that the *g* = 4 signals are observed only in very low temperature spectra.

**Figure 5:**
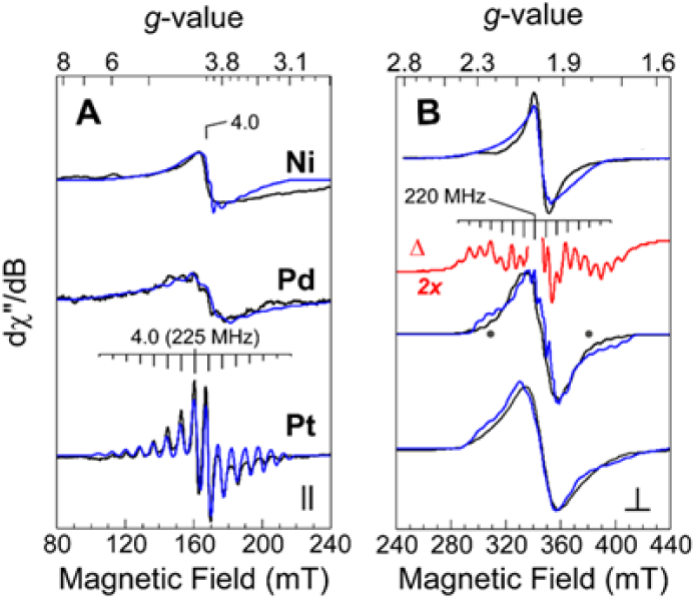
Representative X-band CW EPR spectra collected at 4 K for 10 mM [V ***M*** V] complexes (*M* = Ni, Pd, Pt) with microwave field polarization (*B*_1_) applied parallel (A) and perpendicular (B) to the static magnetic field (*B*_0_). EPR spectroscopic simulations (*blue*) are overlaid on data for comparison. For the Pd complex, a ^51^V multiline hyperfine feature is apparent on the shoulders of the broad *g* ∼ 2 feature, which is more clearly observed by subtracting the broad isotropic derivative from the data (*red trace*, Δ). This multiline feature is reproduced by the simulated spectra in both perpendicular and parallel mode. While absent for the Ni compound, this multiline feature is also present in the Pt spectrum but is less apparent due to line broadening. *Instrumental parameters:* microwave frequency; perpendicular (⊥, 9.46 GHz), parallel (∥, 9.41 GHz) polarization; microwave power, 42 mW (A), 67 μW (B); modulation amplitude, 0.9 mT.

**Figure 6:**
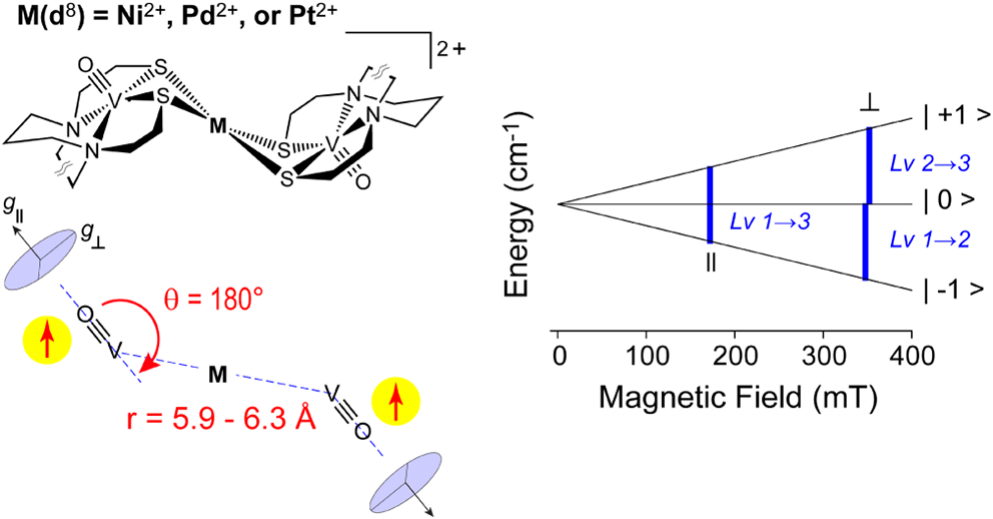
(Left) Schematic of the spin-coupling model for [V**′*M*** V**′**] (*M* = Ni) [V ***M*** V] (*M* = Pd, and Pt) and illustration of the dipolar coupling parameters (*r*, θ) used in EPR simulations. For the simulations shown in Figure 5 and Table 3, *g*- and *A*-tensors were assumed to be collinear. (Right) Energy level diagram of ground state triplet (*S* = 1) illustrating transitions observed in parallel and transverse mode. For simplicity, nuclear splitting from the two ^51^V (*I* = 7/2) sites is omitted.

The most striking difference in the EPR spectra of the [V**′*M*** V**′**] (*M* = Ni) and [V ***M*** V] (*M* = Pd, Pt) compounds is the presence of an intense ^51^V multiline hyperfine feature in the Pt complex split by 225 MHz. This multiline feature confirms spin coupling between two vanadyl ions. For equivalent sites (*N*), the number of lines (15-lines) can be calculated assuming 2*NI*+1 lines given the nuclear spin for ^51^V (*I* = 7/2). Further, the hyperfine splitting observed for a spin-coupled dimer represents a weighted average of the intrinsic hyperfine tensors (*Ã*_s_) for each site projected onto the total spin of the dimer.

5 $${Ã}_{s}=c_{1}\cdot {Ã}_{1}+c_{2}\cdot {Ã}_{2}$$

The projection factors (*c_i_*, where *i* = 1, 2) shown in eq 5 are a function of the intrinsic spin of each site.^36^ However, for equivalent spins (*S*_1_ = *S*_2_ = 1/2), the projection coefficients for each metal are simply *c*_*i*_ = 1/2. Furthermore, often only one hyperfine term in eq 5 is retained when the coupling of spins from one metal site to the adjacent metal nucleus is weak.^36^ Consequently, the magnitude of the observed multiline hyperfine splitting for a dimer of vanadyl ions is half what is observed for an isolated site. While multiline hyperfine features are also observed in parallel mode for the Pd complex, the signal intensity of hyperfine transitions is attenuated >20-fold relative to Pt. Moreover, no hyperfine transitions are observed in parallel mode for the Ni complex.

Perpendicular mode EPR spectra (Figure 5, panel B) for all the complexes **4–6** exhibit a broad derivative signal near *g* ∼ 2.0. This signal arises from overlapping Δ*m*_s_ = ±1 transitions (levels 1→2 and 2→3) within the *S* = 1 manifold as shown in Figure 6 (right). Among the [V ***M*** V]^**2+**^ compounds, the apparent line width follows the trend Ni < Pd < Pt. As in the case of the parallel mode spectra, a multiline feature is observed throughout the broad *g* ∼ 2 signal for the Pd and Pt complexes. For the Pd complex, the ^51^V multiline hyperfine features are most apparent in the shoulders of the spectrum. This can be more clearly observed by subtracting a simulation of the broad isotropic feature from the data (red trace, D). Similar hyperfine features on the transverse mode *g* ∼ 2 signal (albeit broader) are also present in the Pt complex.

The EPR simulations shown in Figure 5 (blue trace) overlaid on experimental data (black) were generated by simultaneously fitting both parallel and transverse mode spectra collected at 4 to 10 K. Table 3 summarizes the spectroscopic parameters used to simulate the EPR data. For all simulations, the experimental modulation amplitude (0.9 mT) was used as the intrinsic EPR line width (*s*_B_). Additional line broadening was simulated by a Gaussian distribution in *g*-values to account for *g*-strain (*s*_g_) along the vanadyl ion principal coordinates. As noted from the data in Table 3, the EPR spectra for the Ni complex is best reproduced assuming ∼2-fold higher *g*-strain relative to the Pd and Pt bridged analogues. This finding is corroborated by a similar 2-to-3-fold increase in thermal β-factors observed in the **[V′NiV′]** (bme-daco) species relative to the Pd- and Pt-[V ***M*** V] (bme-dach) analogues. As the hyperfine splitting for the **[V′NiV′]** ^**2+**^ (bme-daco) cation is unresolved, the magnitude of system hyperfine splitting is inferred from its contribution to the EPR spectral line width. Significantly, both *g*- and *A*-values obtained from simulation of spin-coupled [V ***M*** V]^**2+**^ cations are highly consistent with values reported elsewhere.^59^

**Table 3:** EPR Simulation Parameters for [V ***M*** V]^**2+**^ Complexes^a^

**[V*M*V]** ^**2+**^	**(** *g*_**1**_, ***g***_**2**_, ***g***_**3**_)	**(** *A*_***1***_, ***A***_**2**_, **A**_**3**_ **) [MHz]**	***s*** (*g*_**1**_, ***g***_**2,**_ **g**_**3**_)	***J*** **[cm** ^**–1**^]	*r*, *q*
Ni	1.94, 1.99, 2.11	16, 45, 205	0.01, 0.07, 0.07	0.25	6.01, 180
Pd	1.85, 2.00, 2.10	33, 42, 220	0.01, 0.02, 0.01	0.91	6.27, 180
Pt	1.86, 2.05, 2.10	38, 47, 225	0.02, 0.02, 0.01	1.30	6.22, 180

a For the sake of brevity, only system *G*- and *a*-values are provided rather than the tensors intrinsic to each V-site.

The isotropic and dipolar contributions to the hyperfine tensor (*A*_iso_ and *A*_dip_) were obtained from the following relationships: *A*_iso_ = (*A*_1_ + *A*_2_ + *A*_3_)/3 and *A*_dip_ = *A*_iso_ – *A*_⊥_ = (*A*_*∥*_ – *A*_⊥_)/3.

For all simulations, equivalent and near axially symmetric intrinsic *g*-values are assumed for individual vanadyl sites (*S* = 1/2); the lower *g*_1_ value agrees with the magnetic susceptibility data obtained from SQUID magnetometry. Values used for the Heisenberg–Dirac–van Vleck spin exchange term (***J***) use the Hamiltonian, *Ĥ*_ex_ = −2 ***J*** *S*_1_•*S*_2_. While the magnitude of *J* is too small to accurately determine by fitting of EPR data, the value obtained by SQUID magnetometry accurately predicts changes in the simulated EPR spectral intensity observed within a temperature range of 3.5 to 15 K. The V–O bond vector defines the parallel *g*- and *A*-axis; therefore, the dipolar parameters (*r*, θ) can be taken directly from the crystal structure (Figure 6, left). Values for ϕ were neglected (ϕ = 0) as it was observed to have minimal effect on simulated line shape or intensity.

As noted above, the most dramatic difference in the spectra of the three compounds is the intensity and resolution of ^51^V-hyperfine transitions observed in both parallel and transverse mode polarization, and, to some extent, this can be attributed to increased *g*-strain from structural heterogeneity. However, an additional factor to be considered is electronic and nuclear spin state mixing which also contributes to increased line broadening. This is especially true as the magnitude of electronic and nuclear splitting approach the same value. For example, in the Ni complex, the ratio of electronic exchange relative to *A*_iso_ is ≤83. In contrast, the same ratio is ∼3.3 to 4.5-fold higher for the Pd and Pt complexes, respectively, as noted in Table 4. Consequently, electronic and nuclear spin-state mixing is expected to decrease in going down the group 10 triad, following the trend Ni > Pd > Pt. A 10-fold dilution of samples had no effect on the observed spectra indicating that sample aggregation, or intermolecular spin–spin interactions do not contribute to the spectral line width or attenuation of hyperfine transitions (Figure S19). This observation supports the argument that intramolecular spin–spin interactions (electron–nuclear mixing) is responsible for the attenuated hyperfine pattern observed when ascending group 10 from Pt to Pd to Ni.

**Table 4:** Isotropic and Dipolar Contribution to the ^51^V-Hyperfine Tensor (System *a*-Values)

[V*M*V]^2+^	*A*_*∥*_	*A*_⊥_	*A*_iso_ [MHz]	*A*_dip_ [MHz]	*J/A*_iso_ *(cm* ^*–1*^)
Ni	205	30	90	58	83
Pd	220	40	100	61	275
Pt	225	40	105	61	373

Attempts to collect pulsed EPR data on the Pt–[V*M*V] serial dilutions (shown in Figure S19) were unsuccessful. No detectable echo was observed in the *g* ∼ 2 region within the dead time of the spectrometer over a temperature range of 4–50 K. This rapid electronic relaxation is likely associated with phonon-mediated transitions among thermally accessible spin manifolds.^61–63^

### Computational Studies

Density-functional theory (DFT) calculations were performed using TPSSTPSS functionals to test the agreement of parameters obtained from X-ray diffraction experiments and SQUID magnetometry with theory. Table S4 compares intramolecular distances and metrical parameters determined from DFT calculations (see details in SI) with those taken directly from the X-ray crystal structures of **1**·CH_3_CN, **2**·2CH_3_CN, and **3**·2CH_3_CN. Good agreement between most data points in the three sets indicates that the theory and basis set used to model these molecules are appropriate, although the computer model overpredicted the V–V and V–*M* (*M* = Ni, Pd, and Pt) distances. To investigate the electronic ground state and to evaluate the singlet–triplet energy gaps, calculations were carried out on both the triplet and broken-symmetry (BS) singlet states. Comparing the energy values given by Gaussian shows that, for all three V–*M*–V complexes, the triplet, *S* = 1 state is lower in energy than the *S* = 0 singlet state, by a remarkably similar amount in all three compounds—by 0.651 kcal/mol for **[V′NiV′]** ^**2+**^ and 0.653 kcal/mol for **[VPdV]** ^**2+**^ and **[VPtV]** ^**2+**^. Spin contamination is high in the BS singlet (*S* = 0.627) but almost absent from the ground state triplet, in which case *S* was near 1.01, according to Gaussian. The *J* values in Table S3 were calculated using the equation based on the NP (nonprojected) formula (eq 6):^64^

6 $$J=\frac{\left(E_{LS}-E_{HS}\right)}{2S_{1}S_{2}+S_{2}}$$

where *S*_*1*_ ≥ *S*_*2*_. Computed *J* values follow the same general trend (**[V′NiV′]** ^**2+**^ < **[VPdV]** ^**2+**^ < **[VPtV]** ^**2+**^) found from experimental SQUID data and successfully explain the relative strength of hyperfine coupling found in the parallel EPR spectra of the three compounds. Also, similar to what was observed in the [Fe ***M*** Fe]^**2+**^ cation complexes, the two highest orbitals in the triplet state for all three complexes are the in- and out-of-phase combinations of the α and β open-shell singlet MOs. The energy gap between the triplet HOMO and HOMO–1 is 0.3765 kcal/mol for **[V′NiV′]** ^**2+**^, 0.0251 kcal/mol for **[VPdV]** ^**2+**^, and 1.2989 kcal/mol for **[VPtV]** ^**2+**^ (Figure S25). The **[V’NiV’]** ^**2+**^ cation has a larger gap between its two highest occupied orbitals than **[VPdV]** ^**2+**^, but the different hydrocarbon chain of the N_2_S_2_ ligand may affect the results of the calculation. Molecular orbital diagrams of the compounds (Figure 7; see also Figure S25) show that the HOMO contains the V d_*xy*_ orbital slightly overlapping with the d_*z*_ ^2^ orbital of the Group 10 metal, and that the Group 10 d orbital, as expected, is more diffuse and overlaps more with the vanadium orbitals in **[VPtV]** ^**2+**^ than for **[VPdV]** ^**2+**^ or **[V′NiV′]** ^**2+**^. While the HOMO–1 shows essentially no involvement of the group 10 metal d orbitals for all three compounds (Figure S26), the HOMO–2 has more ligand and central metal contributions than V, and the HOMO–1 to HOMO–2 gap is significantly lower (5.97 kcal/mol) for **[V′NiV′]** ^**2+**^ than for **[VPdV]** ^**2+**^ (20.7 kcal/mol) or **[VPtV]** ^**2+**^ (16.5 kcal/mol), potentially due to the longer hydrocarbon chain of the daco backbone acting to destabilize this orbital.

**Figure 7:**
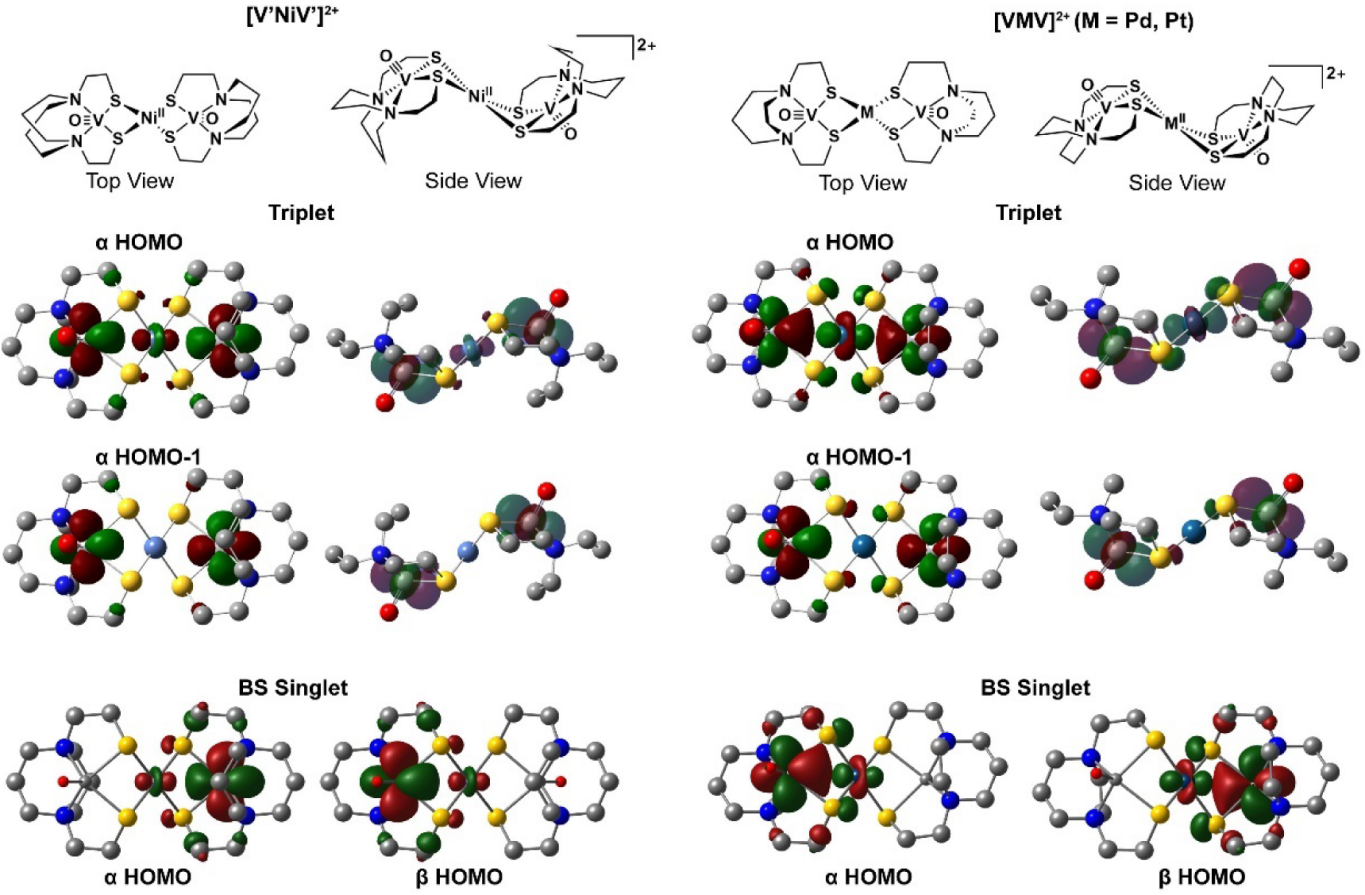
Molecular orbitals of **[V′NiV′]** ^**2+**^, **[VPdV]** ^**2+**^ and **[VPtV]** ^**2+**^ as computed by DFT (TPSSTPSS functional, isovalue 0.03). ChemDraw structures are shown at the top and broken-symmetry (BS) singlet calculations for **[V′NiV′]** ^**2+**^ and **[VPtV]** ^**2+**^ are represented in the figures at bottom. Results of triplet calculations (center) were similar between **[V′NiV′]** ^**2+**^, **[VPdV]** ^**2+**^ and **[VPtV]** ^**2+**^.

## Conclusions

The nature of the bridging unit(s) and ligand donor properties, which influence the structure and occupancy of vanadium energy levels, are key factors governing electronic communication, redox flexibility, magnetic exchange and relaxation times in vanadium oxide-based molecular qubits and conducting materials.^23–27,65^ In addition, the vanadyl unit, (VO)^2+^, has been of interest because of its ability to serve as an EPR spin probe in metalloenzymes such as pyruvate carboxylase when substituted for divalent metals.^59^ It also plays a role in pharmaceuticals including vanadyl sulfate (VOSO_4_), bis-maltolatooxovanadium­(IV) and bis­(ethylmaltolato)­oxovanadium­(IV), proposed for use in treatments for diabetes.^18^ Inspired by naturally occurring metalloenzymes that employ N_2_S_2_-type thiolate backbones, we have synthesized and characterized N_2_S_2_-based [Fe ***M*** Fe] and [V ***M*** V] (*M* = Ni, Pd, Pt) complexes to probe the differences in magnetic behavior between *S* = 1/2 (V=O) and *S* = 1/2 {Fe­(NO)}^7^ thiolate-bridged systems. Both series have essentially the same spin configuration and stair-step geometry, but the unpaired electron resides in a d_*z*_^2^ orbital for {Fe­(NO)}^7^),^16^ while for vanadyl it is in a d_*xy*_ orbital which does not have the σ symmetry required to overlap with the S 3p_*z*_ orbitals.

Based on earlier studies with N_2_S_2_-based [Fe ***M*** Fe] diradical complexes (*M* = Ni, Pd, Pt) we expected that the magnitude of the ferromagnetic coupling would increase in going down the triad. Indeed, experimental and calculated values of *J* for ferromagnetic coupling in the VO­(N_2_S_2_)-*M*- VO­(N_2_S_2_) analogues were found to vary in the order *M* = Pt > Pd > Ni, although they were much weaker than the antiferromagnetic coupling observed for the Fe series (Figure S14). This result is accounted for by the fact that the orbital involvement that controls the coupling is completely different. The molecular orbitals from DFT calculations indicate that the contribution of the Pt 5d orbital to the HOMO of **[VPtV]** ^**2+**^ is greater than are the Pd-4d or Ni-3d contributions to the HOMO of **[VPdV]** ^**2+**^ and **[V′NiV′]** ^**2+**^. The more diffuse Pd and Pt orbitals promote a stronger magnetic coupling as they have increased overlap with the 3p orbitals in the soft sulfur bridge. Magnetic susceptibility measurements demonstrate a weak ferromagnetic coupling between the V^IV^ spin centers over distances of 5.9–6.3 Å. The observed sign of the magnetic coupling constant agrees with orthogonality arguments and the Goodenough–Kanamori rules.^6,7^ These results suggest that heavier elements hold promise for the design of bridged multimetallic molecules with stronger magnetic coupling, a pattern also observed in cyanide-bridged 3d–5d complexes^66^ and other compounds where iron­(II) is bridged by a group 10 metal.^67^ In the EPR spectra, the 15-line hyperfine pattern, observed for **[VPdV]** ^**2+**^ and **[VPtV]** ^**2+**^ at low temperature, demonstrates that the two vanadyl spin centers (*I* = 7/2) are spin coupled to each other. The weaker coupling between the VO groups, together with possible broadening from zero-field splitting and/or mixing with nuclear spin states, led to the ^51^V coupling pattern being severely broadened for **[VPdV]** ^**2+**^ and not observed at all for **[V′NiV′]** ^**2+**^.

Overall, these results establish N_2_S_2_-based thiolate scaffolds as promising platforms for tuning spin–spin interactions over long distances. Future studies employing more electron-donating N_2_S_2_ ligands, such as *N*,*N*′-ethylene-bis­(mercaptoethylacetamide) (ema) or gem-dimethyl derivatives and incorporating heavier group 11 metal ions such as Ag^I^ and Au^I^ may further enhance coupling and expand the design space for bioinspired molecular architectures with potential in spintronics and quantum information science.

## Supplementary Material



## Notes

Mass spectra, additional magnetic susceptibility figures, crystal and refinement data, packing diagrams, selected bond lengths and angles (PDF)
