# Supplementary material for: Magnetic and EPR Spectroscopic Studies of Thiolate Bridged Divalent Ni, Pd, and Pt Ions Capped with VO(N 2 S 2 ) Metalloligands
Source: Inorg Chem. 2026 Jan 21;65(4):2361–73. doi: 10.1021/acs.inorgchem.5c05204 (PMC12869484; doi:10.1021/acs.inorgchem.5c05204)
Supplement: Supplementary file 1 [file ic5c05204_si_001.pdf]

Supporting Information

Magnetic and EPR Spectroscopic Studies of Thiolate Bridged Divalent Ni, Pd and Pt Ions Capped with $VO(N_2S_2)$ Metalloligands

Dakota D. Jones,† Manuel Quiroz,† Aruzhan Abdikaiym,‡ Akhil K. Singh, Naushad Ahmed, Brad S. Pierce,‡ Marcetta Y. Darensbourg*† and Kim R. Dunbar*†

†*Department of Chemistry Texas A&M University, College Station, TX 77843*
‡ *Department of Chemistry & Biochemistry, University of Alabama, Tuscaloosa, Alabama 35487, USA*

Table of Contents

Synthesis of the $BArF_{24}^-$ analogues 4, 5 and 6 (**$BArF_{24}^-$** = tetrakis((3,5-trifluoromethyl)phenyl)borate........S4-S5

Scheme S1**.** Salt metathesis reaction for synthesis of $BArF_{24}^-$ analogues **4, 5** and **6**........S5

Figure S1. Positive-ion ESI-MS data for **$[V'NiV']^{2+}$** and isotopic bundle for the parent ion peak (inset), collected in CH_3CN........S6

Figure S2. Positive-ion ESI-MS data for **$[VPdV]^{2+}$** and isotopic bundle for the parent ion peak (inset), collected in CH_3CN........S6

Figure S3. Positive-ion ESI-MS data for **$[VPtV]^{2+}$** and isotopic bundle for the parent ion peak (inset), collected in CH_3CN........S7

Figure S4. Electronic spectra of 1, 2 and 3 in MeCN, measured in dilute solutions to observe absorptions in the UV (top) and concentrated solutions in the visible spectrum (bottom)........S8

Figure S5. Cyclic voltammogram of **4** in CH_3CN at 100 mV/s........S9

Figure S6. Cyclic voltammogram of **5** in CH_3CN at 100 mV/s........S9

Figure S7. Cyclic voltammogram of **6** in CH_3CN at 100 mV/s........S10

Figure S8. Scan rate dependence of **5** in CH_3CN........S10

Figure S9. Cyclic voltammogram of VO(bme-daco) in CH_3CN at 100 mV/s........S11

Figure S10. Cyclic voltammogram of VO(bme-dach) in CH_3CN at 100 mV/s........S11

Figure S11. Cyclic voltammogram of $[Ni(MeCN)_6](BF_4)_2$ in CH_3CN at 100 mV/s........S12

Figure S12. Cyclic voltammogram of $[Pd(MeCN)_4](BF_4)_2$ in CH_3CN at 100 mV/s........S12

Figure S13. Cyclic voltammogram of $[Pt(MeCN)_4](BF_4)_2$ in CH_3CN at 100 mV/s........S13

Figure S14. Comparison of X_mT vs T data for $[FeMFe](BF_4)_2$[17] and $[VMV](BF_4)_2$ compounds........S14

Figure S15. Curie-Weiss fits of the magnetic susceptibility data for complexes **1-3**, plotted as $\chi_M = f(T)$ and $\chi_M^{-1} = f(T)$....S15

Figure S16. $\chi_m T$ vs. T plots, at 0.1 T, of **[V'NiV'](BF$_4$)$_2$ (1)** (top, green circles), **[VPdV](BF$_4$)$_2$ (2)** (middle, orange circles), and **[VPtV](BF$_4$)$_2$ (3)** (bottom, purple circles) (circles: raw data, line: fit). Solid lines are the fits given by PHI. Fit parameters: **1**: g = 1.91, J = 0.258 cm^{-1}, TIP = 1.17 * 10^{-3} emu*mol, **2**: g = 1.89, J = 1.064 cm^{-1}, TIP = 0.423*10^{-3} emu*mol, **3**: g = 1.95, J = 1.374 cm^{-1}, TIP = 1.73*10^{-3} emu*mol. The discontinuity in the graph of **2** is due to the sample bag shifting in the holder....S16

Figure S17. χ_m vs. T plots of **[V'NiV'](BF$_4$)$_2$ (1)** (top), **[VPdV](BF$_4$)$_2$ (2)** (middle), and **[VPtV](BF$_4$)$_2$ (3)** (bottom) measured at 1 T (circles: raw data, line: fit)....S17

Figure S18. M vs. H plots of **[V'NiV'](BF$_4$)$_2$ (1)** (top), **[VPdV](BF$_4$)$_2$ (2)** (middle), and **[VPtV](BF$_4$)$_2$ (3)** (bottom) measured from 2-5 K and 0-7 T. S18

Table S1. Fits of magnetic susceptibility data of **1, 2** and **3** at 1 T with spin Hamiltonian (SH) parameters *g* and *J* varied by ±0.01....S19

Figure S19. Perpendicular (⊥) and parallel (||) mode CW X-band EPR spectra for serial dilutions of **[VPtV](BArF$_{24}$)$_2$ (6)**. S20

Table S2. Crystal Data and Structure Refinement for **[V'NiV'](BF$_4$)$_2$ (1), [VPdV](BF$_4$)$_2$ (2),** and **[VPtV](BF$_4$)$_2$ (3)**....S21

Table S3. Crystal Data and Structure Refinement for **[V'NiV'](BArF$_{24}$)$_2$ (4), [VPdV](BArF$_{24}$)$_2$ (5)** and **[VPtV](BArF$_{24}$)$_2$ (6)**....S22

Table S4. Experimental and computed metrical parameters for **[V'NiV']$^{2+a}$**, **[VPdV]$^{2+}$**, and **[VPtV]$^{2+}$**....S23

Table S5. Selected Bond Lengths for **[V'NiV'](BF$_4$)$_2$ (1)**....S24

Table S6. Selected Bond Lengths for **[VPdV](BF$_4$)$_2$ (2)**....S24

Table S7. Selected Bond Lengths for **[VPtV](BF$_4$)$_2$. (3)**....S24

Table S8. Bond Angles for **[V'NiV'](BF$_4$)$_2$ (1)**....S25-S26

Table S9. Bond Angles for **[VPdV](BF$_4$)$_2$ (2)**....S26-S27

Table S10. Bond Angles for **[VPtV](BF$_4$)$_2$ (3)**....S28-S29

Figure S20. N_2S_2 (red) and MS_4 (green) best planes calculated through Mercury for **1**, **2** and **3**....S30

Figure S21. Crystal packing diagram of **[V'NiV'](BF$_4$)$_2$ (1)** looking down the *b* axis....S31

Figure S22. Crystal packing diagram of **[VPdV](BF$_4$)$_2$ (2)** looking down the *a* axis....S32

Figure S23. Crystal packing diagram of **[VPtV]$(BF_4)_2$ (3)** looking down the *a* axis........................S33

Figure S24. Powder X-ray diffraction patterns of **1, 2** and **3** compared to Mercury simulations..,S34

Figure S25. Alpha and Beta MO energy levels from HOMO-4 to LUMO+4 for the triplet (ground) state of the cations in the **[VMV]$^{2+}$** series..S35

Figure S26. Alpha HOMO and HOMO-1 surface density plots (isovalue = 0.03) for the **[VMV]$^{2+}$** cation series..S35

Computational Coordinates (Optimized triplet) **[V'NiV']$^{2+}$ (1)**..S36
Computational Coordinates (Optimized triplet) **[VPdV]$^{2+}$ (2)**..S37
Computational Coordinates (Optimized triplet) **[VPtV]$^{2+}$ (3)**..S38

Synthesis of the $BArF_{24}^-$ analogues 4, 5 and 6 ($BArF_{24}^-$ = tetrakis((3,5-trifluoromethyl)phenyl)borate)

***[VO(bme-daco)-Ni^{II}-VO(bme-daco)]($BArF_{24}$)$_2$*, [V'-Ni-V']($BArF_{24}$)$_2$. (4)** In a 20 mL vial, compound **1** (29.5 mg, 0.035 mmol) was dissolved in 5 mL of CH_3CN. A sample of $KBArF_{24}$ (64.2 mg, 0.071 mmol) was then added to the solution, which was stirred at room temperature for 16 h under Ar; the CH_3CN was evaporated by blowing a stream of Ar over the mixture. The resulting brown solid was dissolved in CH_2Cl_2 (4 mL) and filtered through a small Celite® plug, to give a purple filtrate solution. Red-violet needle crystals of **4•2CH_2Cl_2** (50.2 mg, 59%) were grown by layering hexanes on the filtered CH_2Cl_2 solution at room temperature.

***[VO(bme-dach)-Pd^{II}-VO(bme-dach)]($BArF_{24}$)$_2$*, [V-Pd-V]($BArF_{24}$)$_2$. (5)** In a 20 mL vial, compound **2** (37.3 mg, 0.053 mmol) and $NaBArF_{24}$ (90.4 mg, 0.053 mmol) were combined and dissolved in 6 mL of CH_3CN. The mixture was stirred at room temperature for 14 h under N_2 and the CH_3CN was removed by passing a stream of N_2 over the solution. The resulting light green solid was dissolved in CH_2Cl_2 (4 mL) and filtered through a small Celite® plug. Green needle-like crystals of **5•2CH_2Cl_2** were grown by layering pentane on the filtered CH_2Cl_2 solution at room temperature.

***[VO(bme-dach)-Pt^{II}-VO(bme-dach)]($BArF_{24}$)$_2$*, [V-Pt-V]($BArF_{24}$)$_2$. (6)**. In separate vials, VO(bme-dach) (15.2 mg, 0.053 mmol) was suspended in 2 mL of CH_3CN and [Pt^{II}(CH_3CN)$_4$](BF_4)$_2$ (13.0 mg, 0.024 mmol) was dissolved in 1 mL of CH_3CN. The Pt^{II}(CH_3CN)$_4$(BF_4)$_2$ solution was added dropwise to the VO(bme-dach) suspension, and the mixture was stirred at room temperature for 16 h under N_2. The resulting brownish-green solution was filtered through a small Celite® plug

and $NaBArF_{24}$ (47.7 mg, 0.053 mmol) added to the pale green filtrate. The mixture was stirred at room temperature for an additional 4.5 h under N_2 and the CH_3CN was removed by blowing a stream of Ar over the mixture to give a brownish-green solid which was dissolved in CH_2Cl_2 (3 mL) and filtered through a small Celite® plug. Brown-green needle-like X-ray quality crystals of **6·2CH₂Cl₂** were obtained by layering pentane on the orange CH_2Cl_2 solution at room temperature.

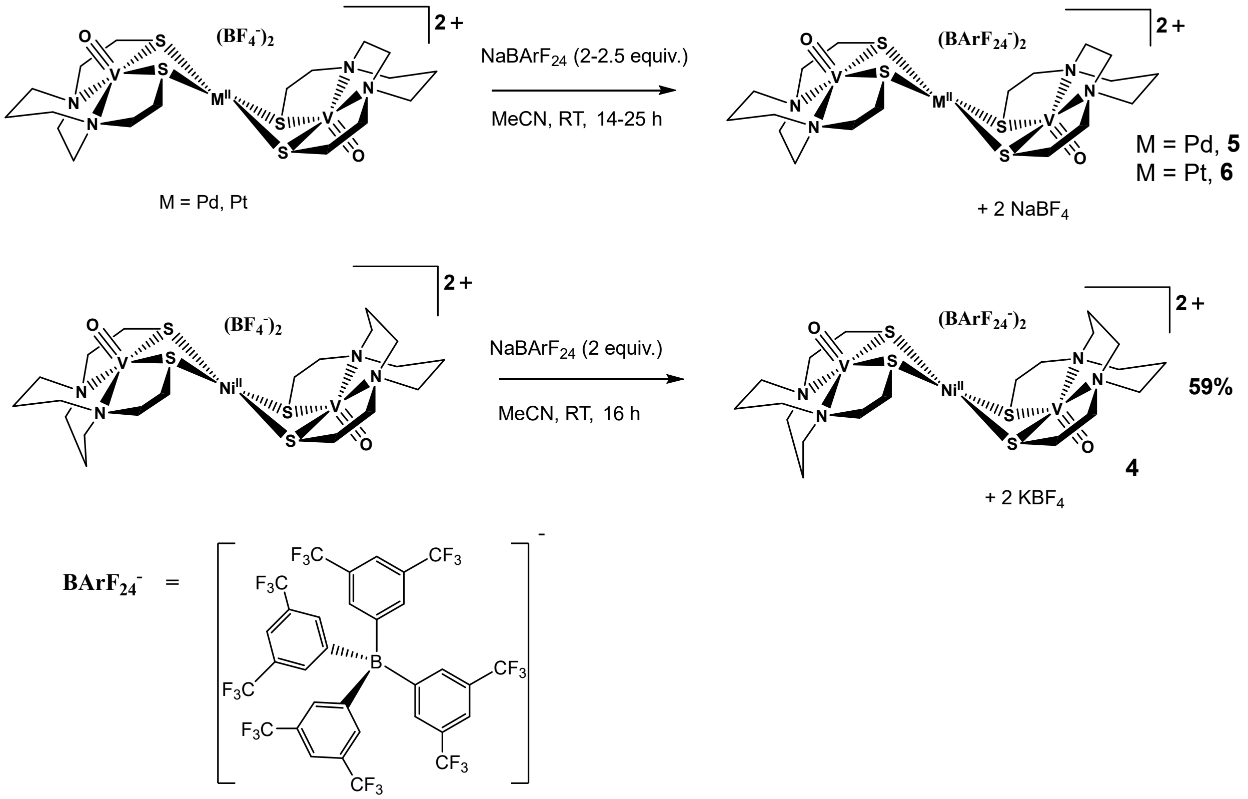

Scheme S1. Salt metathesis reaction for synthesis of $BArF_{24}^{-}$ analogues **4, 5** and **6.**

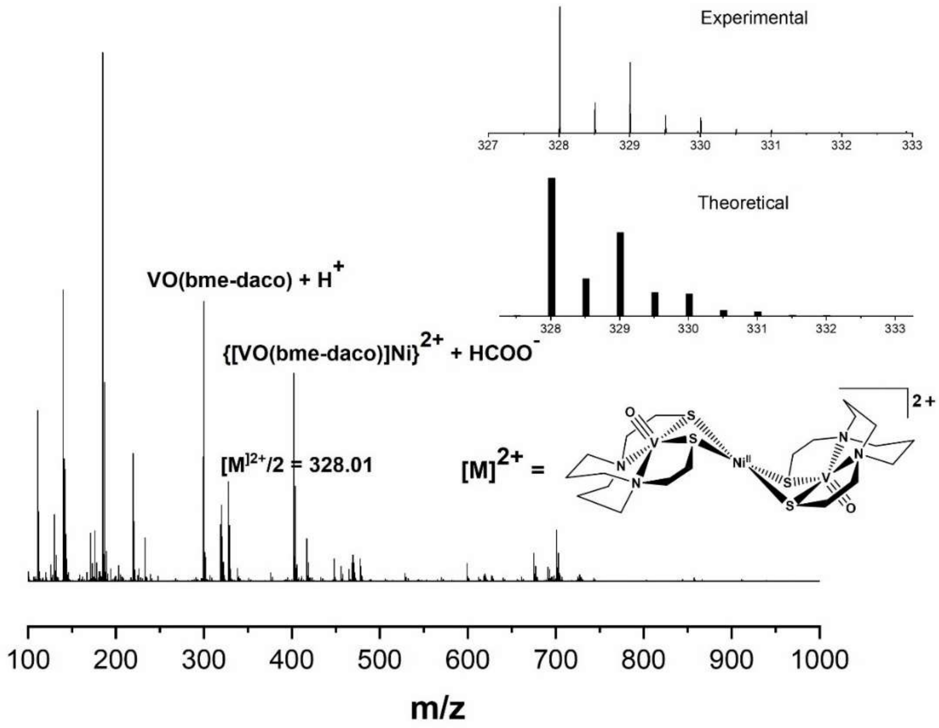

Figure S1. Positive-ion ESI-MS data for **[V’NiV’]$^{2+}$** and isotopic bundle for the parent ion peak (inset), collected in CH_3CN.

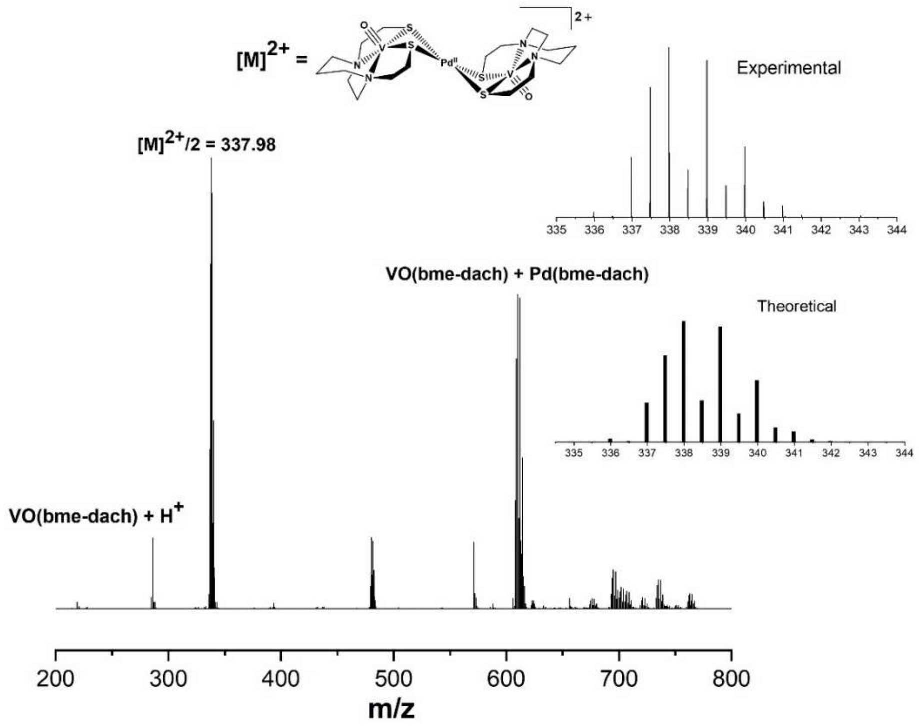

Figure S2. Positive-ion ESI-MS data for **[VPdV]$^{2+}$** and isotopic bundle for the parent ion peak (inset), collected in CH_3CN.

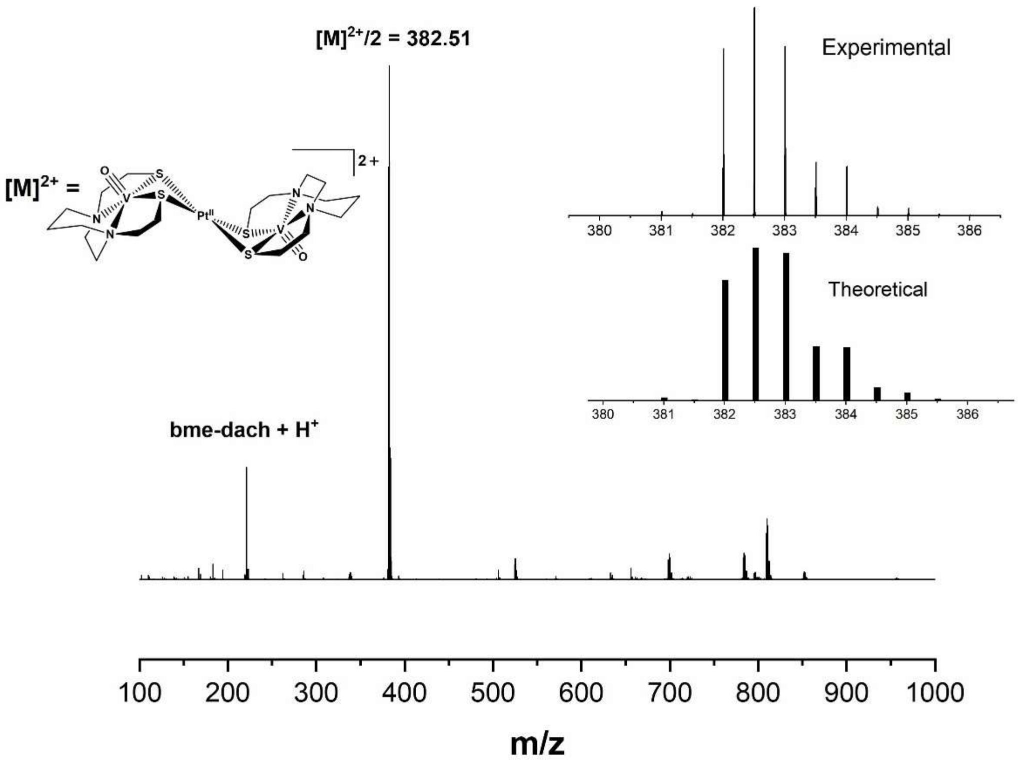

Figure S3. Positive-ion ESI-MS data for **[VPtV]$^{2+}$** and isotopic bundle for the parent ion peak (inset), collected in CH_3CN.

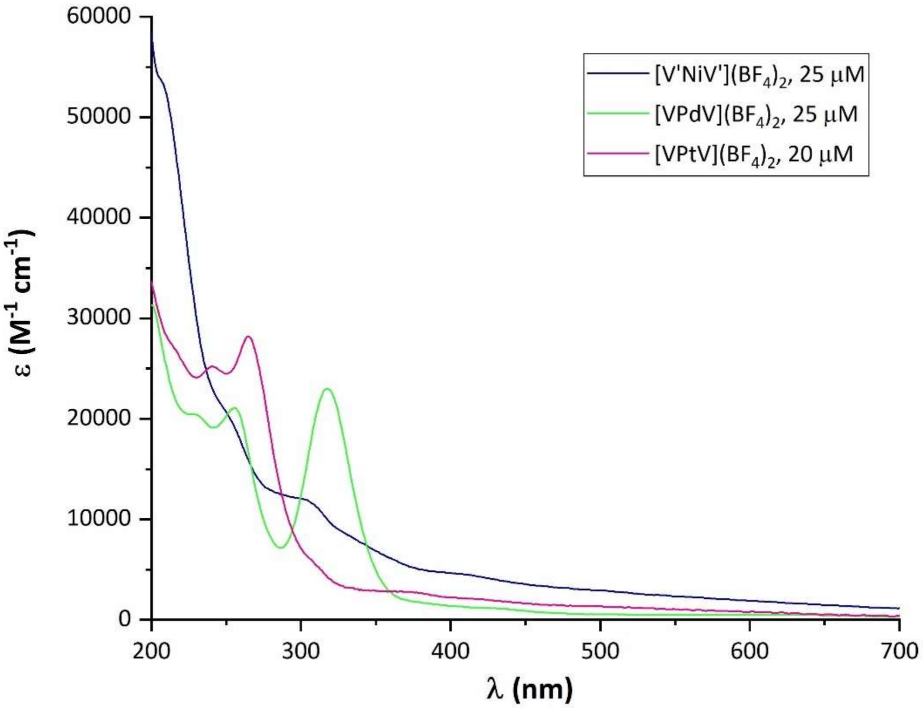

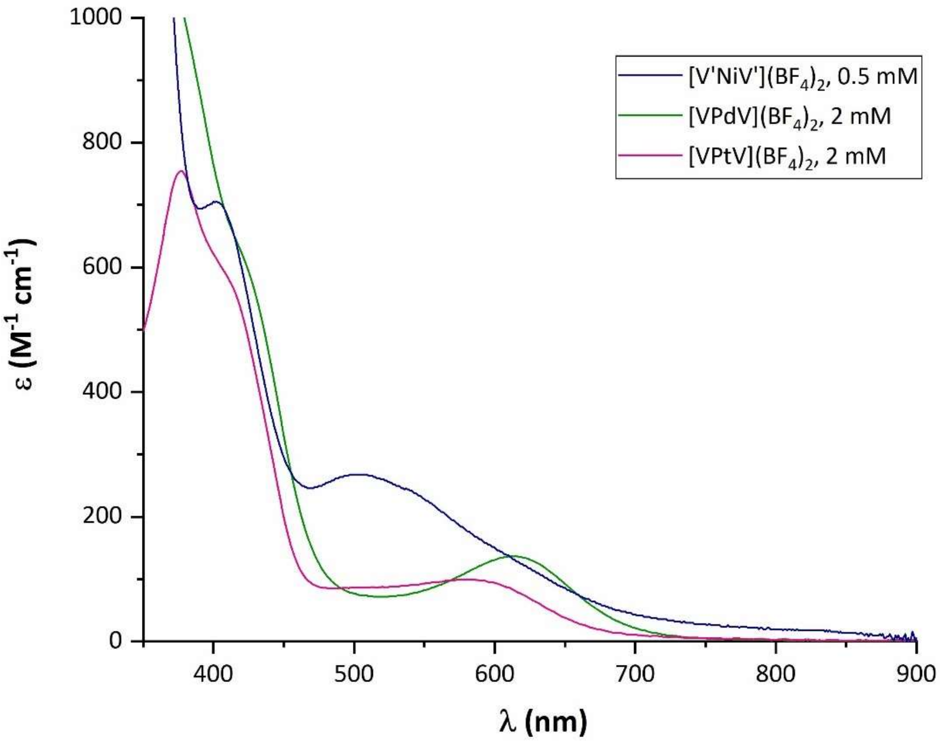

Figure S4. Electronic spectra of 1, 2 and 3 in MeCN, measured in dilute solutions to observe absorptions in the UV (top) and concentrated solutions in the visible spectrum (bottom).

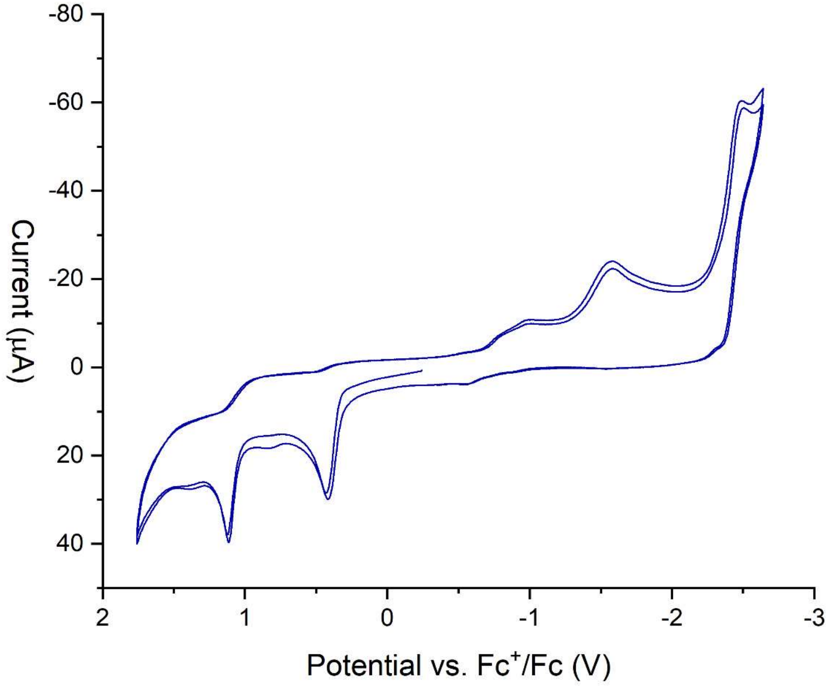

Figure S5. Cyclic voltammogram of **4** in CH_3CN at 100 mV/s.

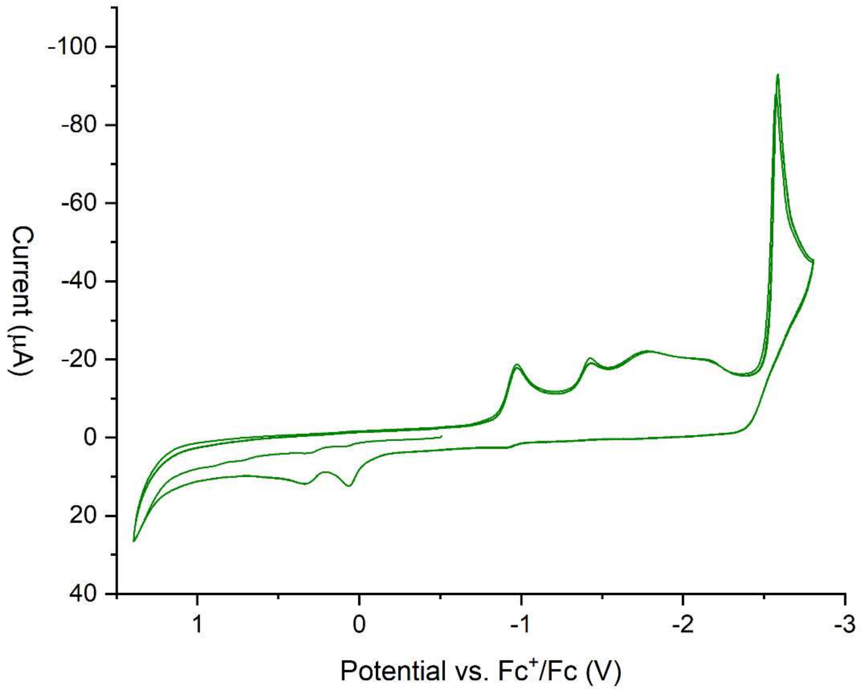

Figure S6. Cyclic voltammogram of **5** in CH_3CN at 100 mV/s.

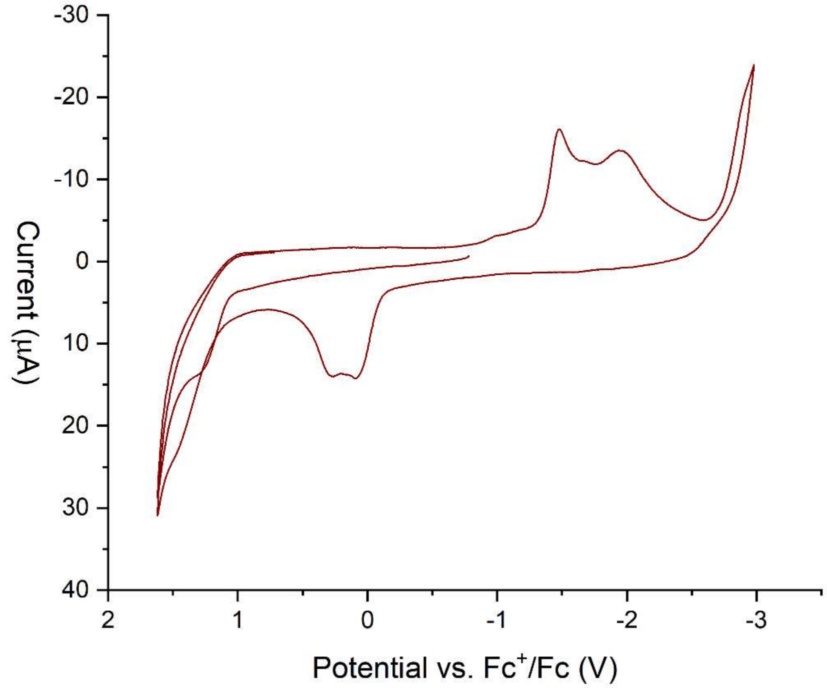

Figure S7. Cyclic voltammogram of **6** in CH_3CN at 100 mV/s.

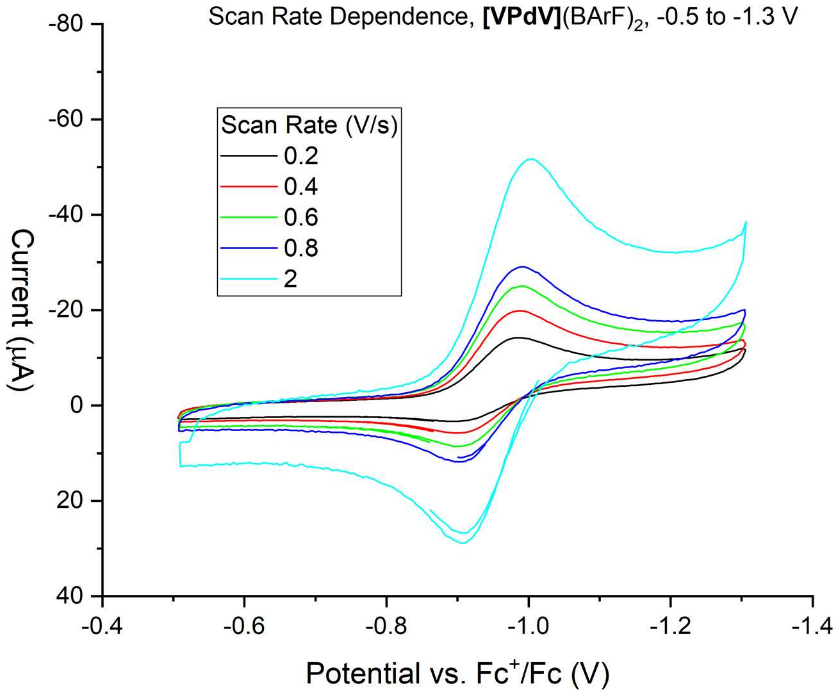

Figure S8. Scan rate dependence of **5** in CH_3CN.

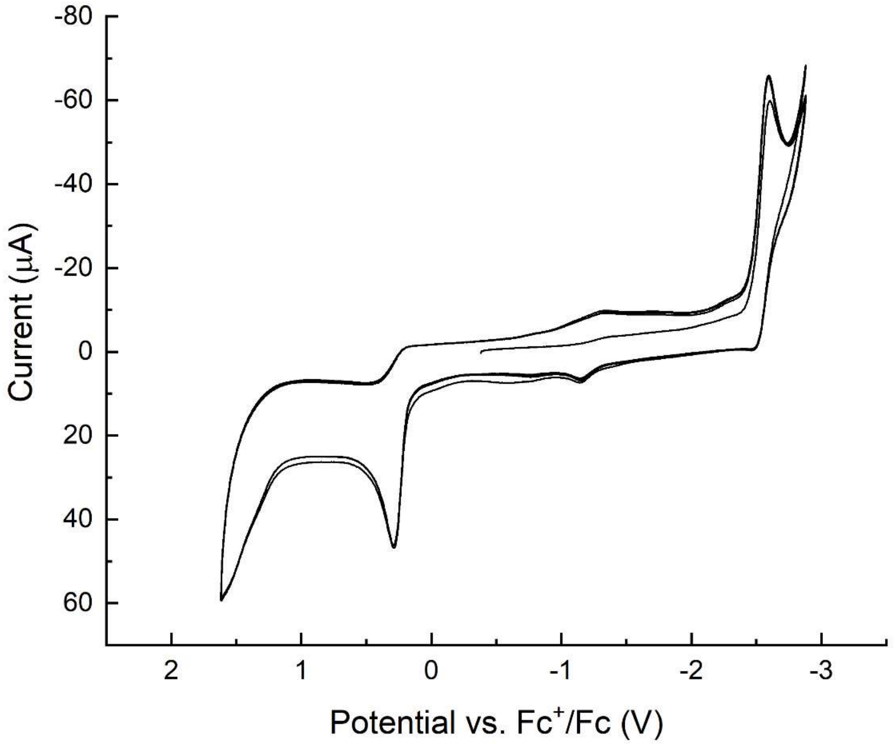

Figure S9. Cyclic voltammogram of VO(bme-daco) in CH_3CN at 100 mV/s.

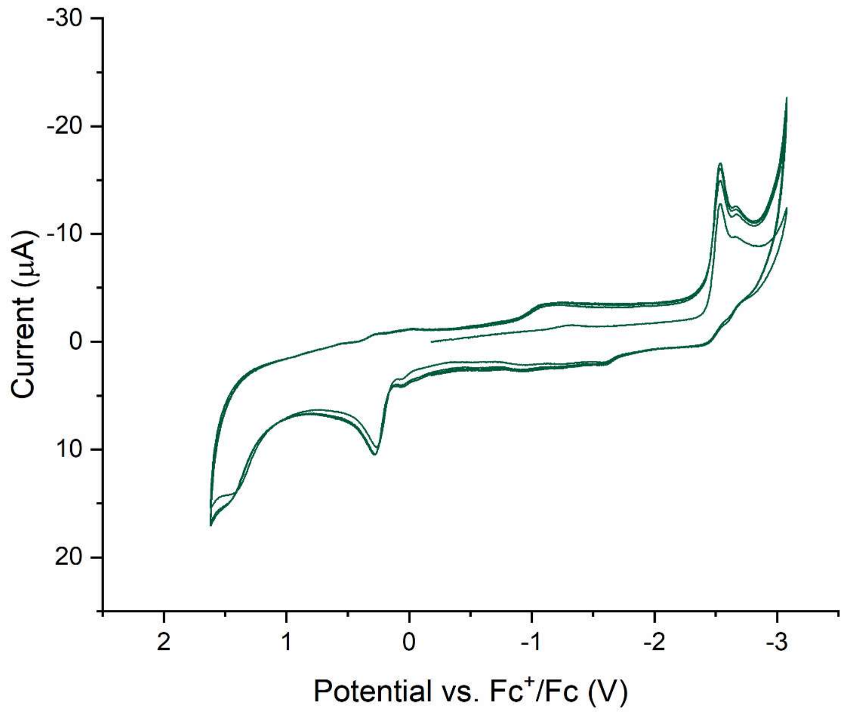

Figure S10. Cyclic voltammogram of VO(bme-dach) in CH_3CN at 100 mV/s.

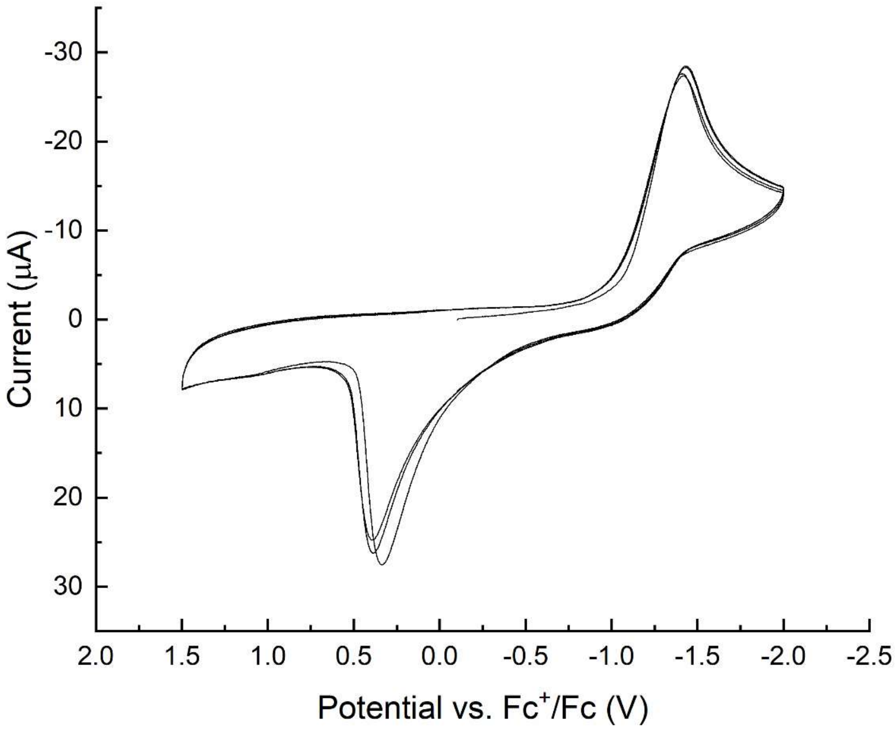

Figure S11. Cyclic voltammogram of $[Ni(MeCN)_6](BF_4)_2$ in CH_3CN at 100 mV/s.

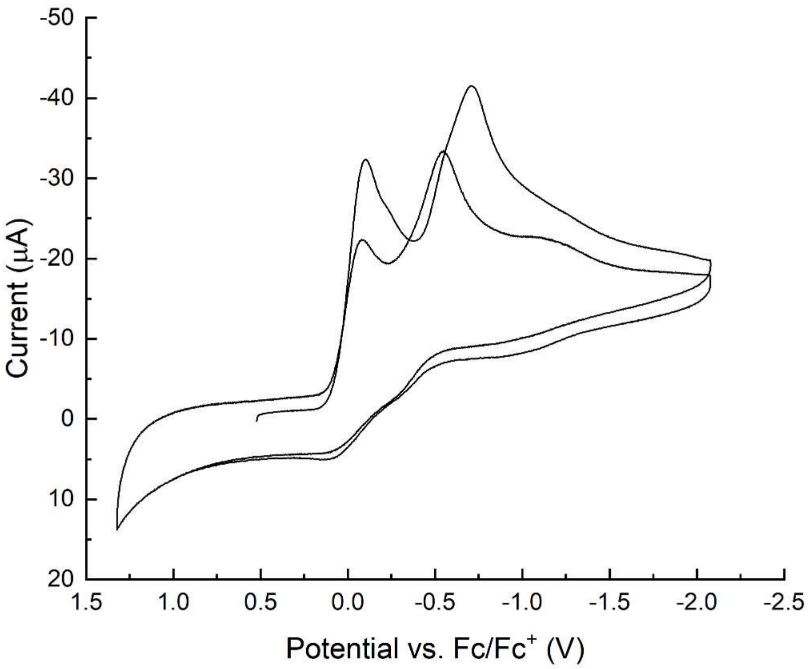

Figure S12. Cyclic voltammogram of $[Pd(MeCN)_4](BF_4)_2$ in CH_3CN at 100 mV/s.

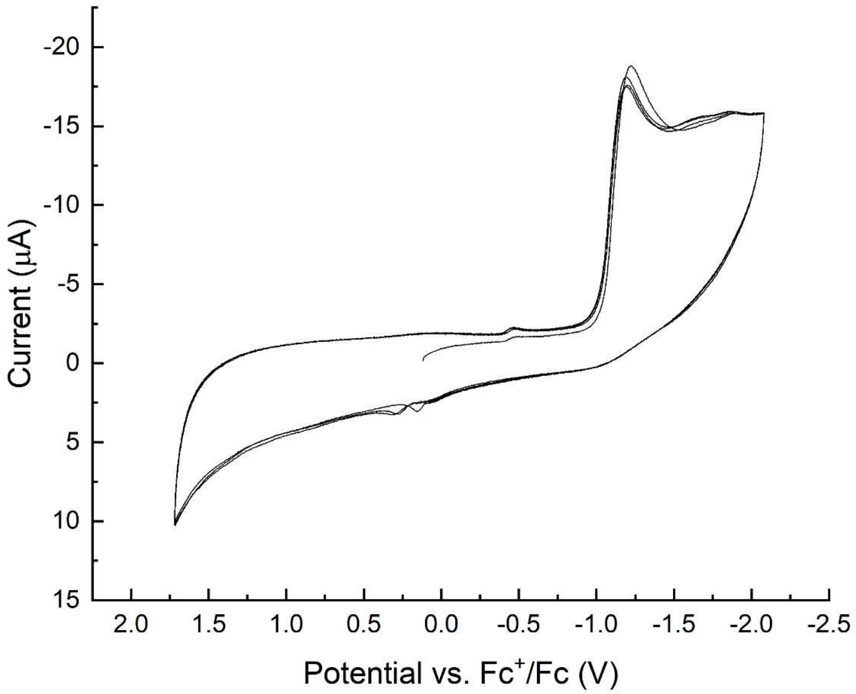

Figure S13. Cyclic voltammogram of $[Pt(MeCN)_4](BF_4)_2$ in CH_3CN at 100 mV/s.

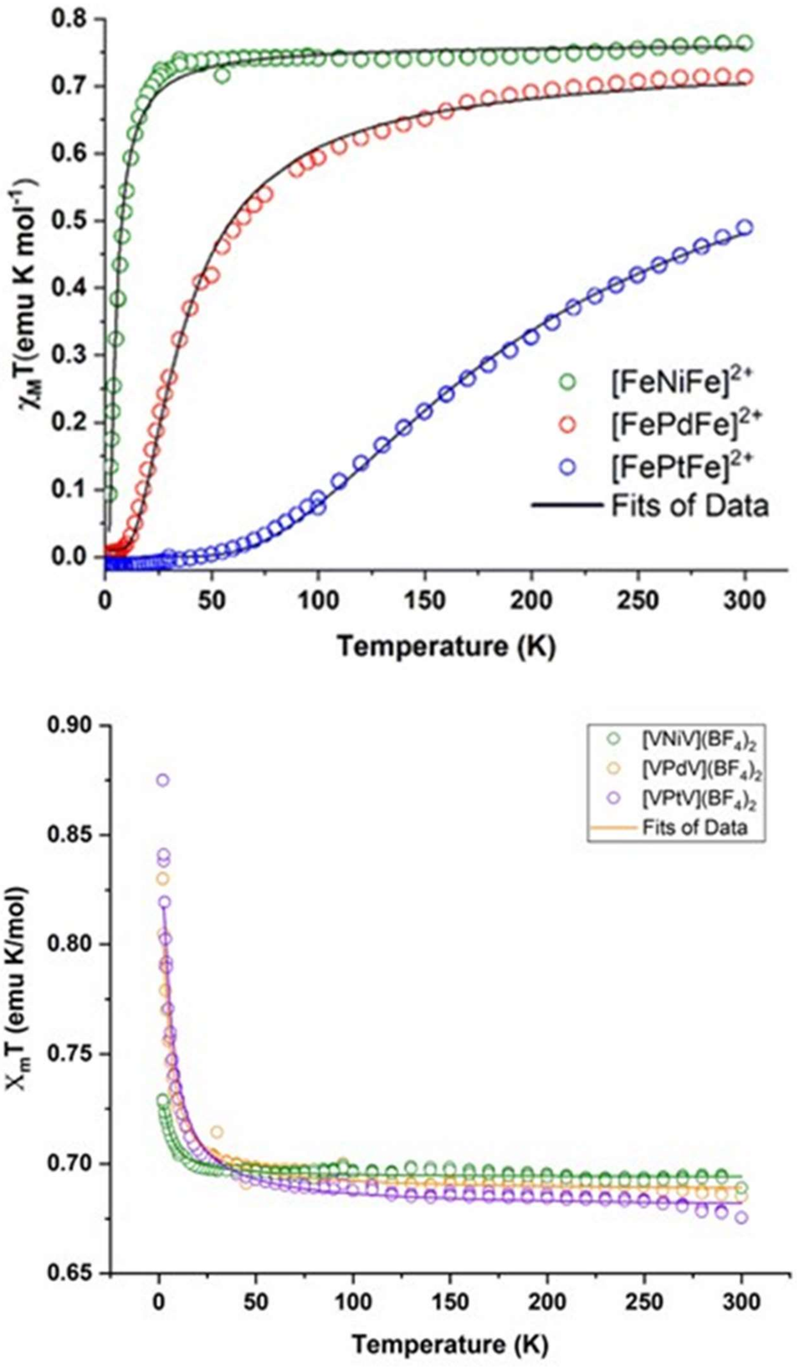

Figure S14. Comparison of χ_mT vs T data for [FeMFe](BF_4)$_2$ and [VMV](BF_4)$_2$ compounds. Top graph reproduced from ref. 16. Available under a CC-BY NC 3.0 license. Copyright 2023 Manuel Quiroz, Molly M. Lockart, Shan Xue, Dakota Jones, Yisong Guo, Brad S. Pierce, Kim R. Dunbar, Michael B. Hall, Marcetta Y. Darensbourg.

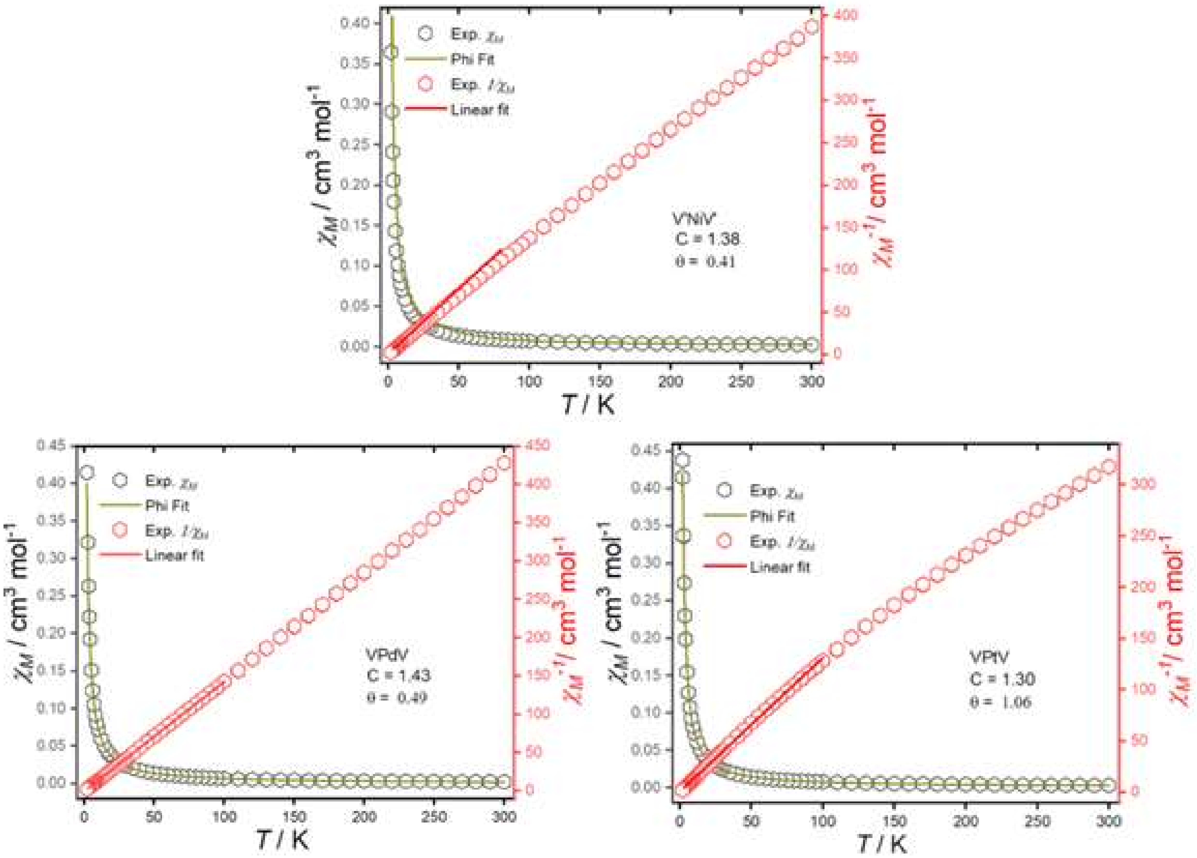

Figure S15. Curie-Weiss fits of the magnetic susceptibility data for complexes **1-3**, plotted as χ_M = $f(T)$ and χ_M^{-1} = $f(T)$.

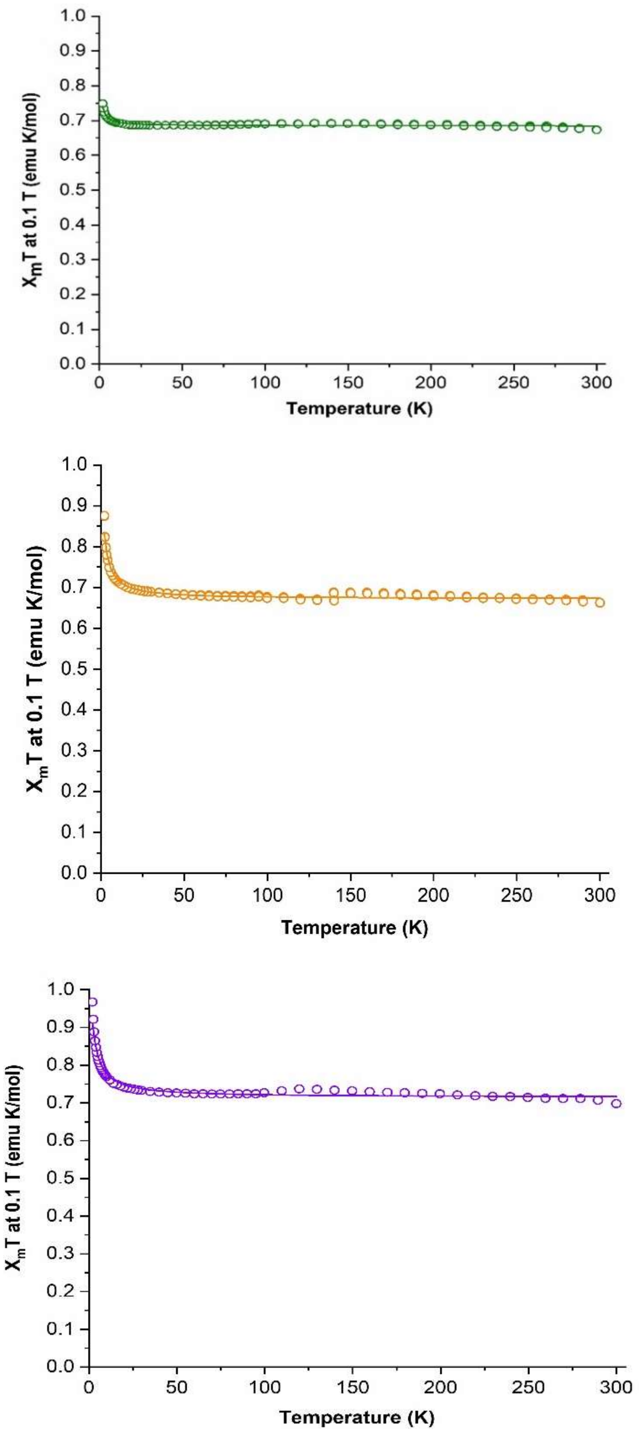

Figure S16. $\chi_m T$ vs. T plots, at 0.1 T, of **[V'NiV'](BF$_4$)$_2$ (1)** (top, green circles), **[VPdV](BF$_4$)$_2$ (2)** (middle, orange circles), and **[VPtV](BF$_4$)$_2$ (3)** (bottom, purple circles) (circles: raw data, line: fit). Solid lines are the fits given by PHI. Fit parameters: **1**: g = 1.91, J = 0.258 cm^{-1}, TIP = 1.17 * 10^{-3} emu*mol, **2**: g = 1.89, J = 1.064 cm^{-1}, TIP = 0.423*10^{-3} emu*mol, **3**: g = 1.95, J = 1.374 cm^{-1}, TIP = 1.73*10^{-3} emu*mol. The discontinuity in the graph of **2** is due to the sample bag shifting in the holder.

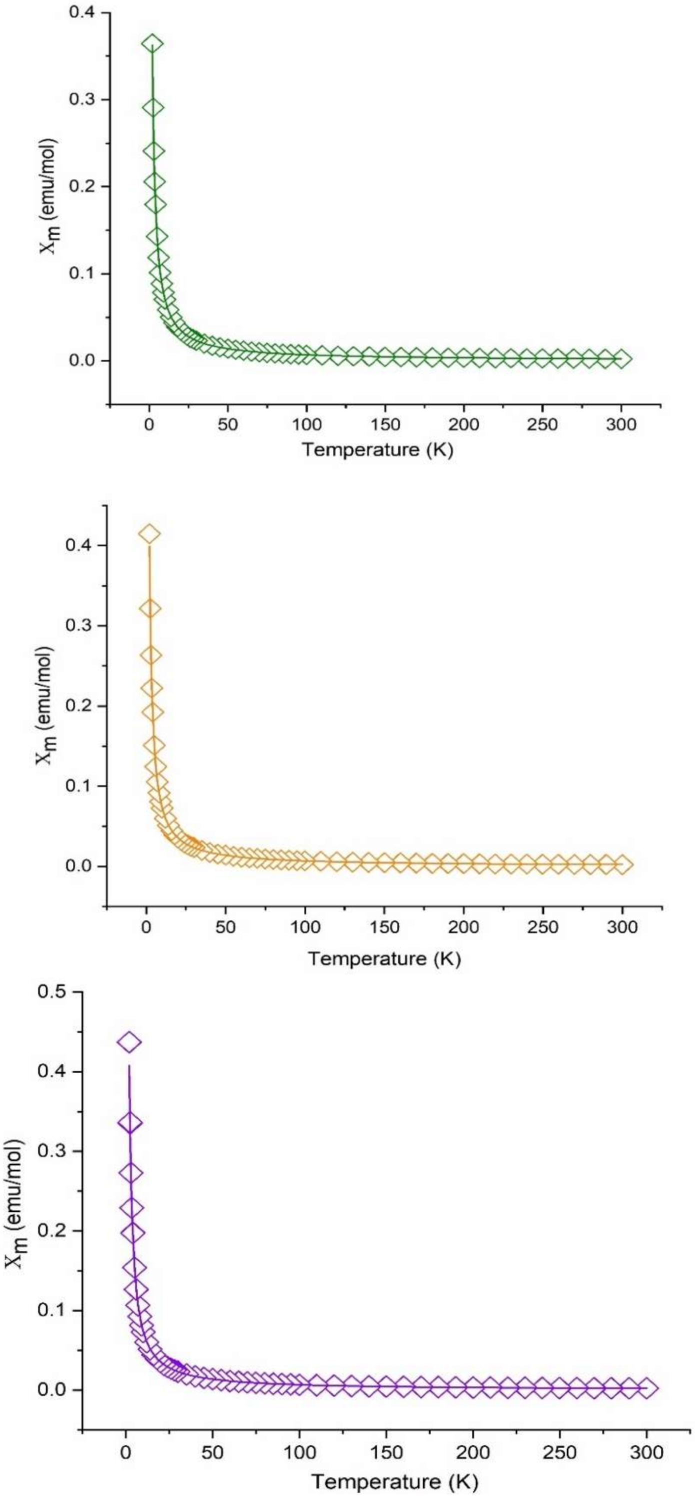

Figure S17. χ_m vs. T plots of **[V'NiV'](BF$_4$)$_2$ (1)** (top), **[VPdV](BF$_4$)$_2$ (2)** (middle), and **[VPtV](BF$_4$)$_2$ (3)** (bottom) measured at 1 T (circles: raw data, line: fit).

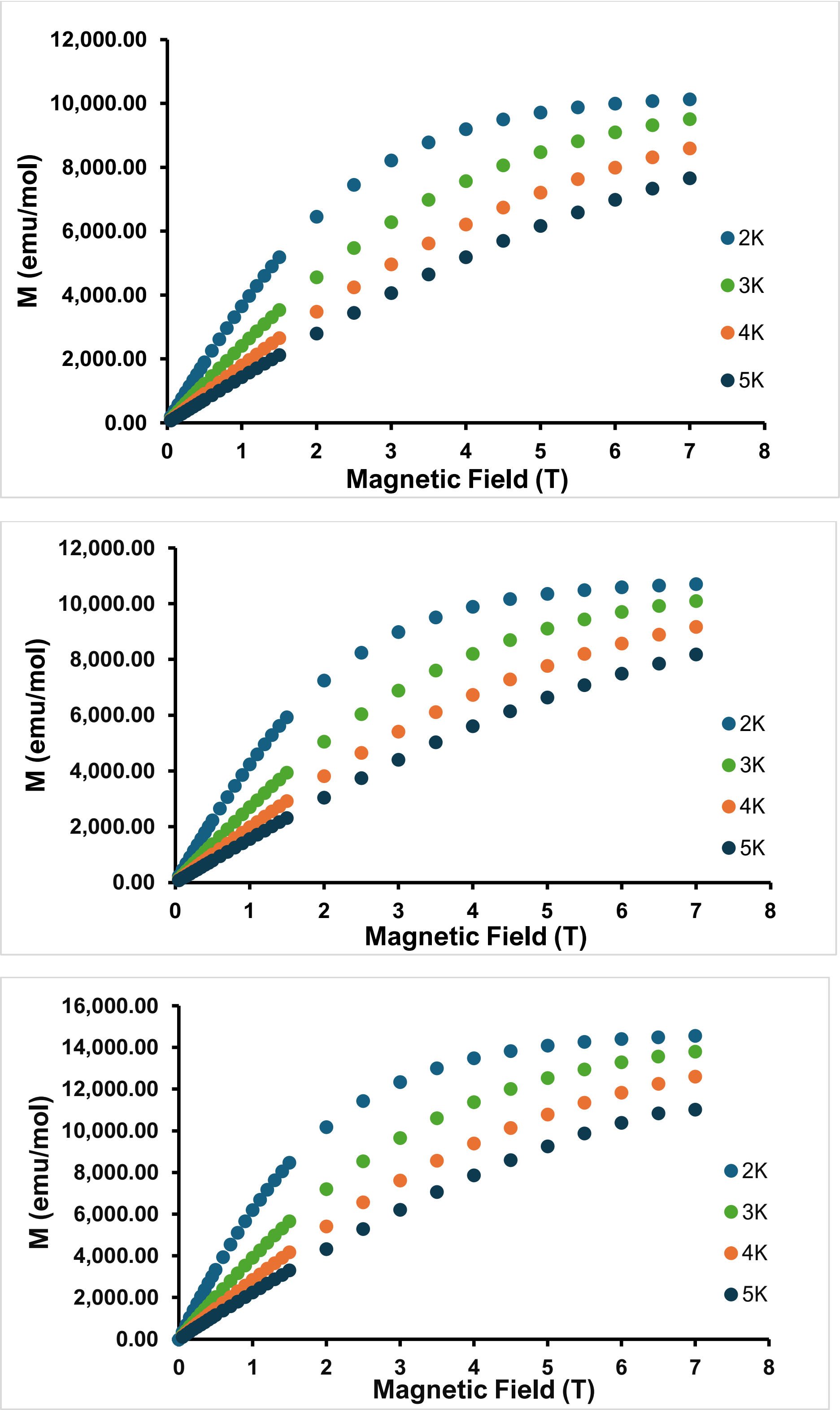

Figure S18. M vs. H plots of **[V'NiV'](BF$_4$)$_2$ (1)** (top), **[VPdV](BF$_4$)$_2$ (2)** (middle), and **[VPtV](BF$_4$)$_2$ (3)** (bottom) measured from 2-5 K and 0-7 T.

Table S1. Fits of magnetic susceptibility data of **1, 2** and **3** at 1 T with spin Hamiltonian (SH) parameters *g* and *J* varied by ±0.01.

Complex	SH parameter (input)	PHI Outcome
**[V'NiV'](BF$_4$)$_2$**	J = 0.292 cm^{-1}	g = 1.923
	J = 0.272 cm^{-1}	g = 1.924
	g = 1.93	J = 0.226 cm^{-1}
	g = 1.91	J = 0.399 cm^{-1}
**[VPdV](BF$_4$)$_2$**	J = 0.964 cm^{-1}	g = 1.914
	J = 0.944 cm^{-1}	g = 1.914
	g = 1.92	J = 0.878 cm^{-1}
	g = 1.90	J = 1.182 cm^{-1}
**[VPtV](BF$_4$)$_2$**	J = 1.382 cm^{-1}	g = 1.903
	J = 1.362 cm^{-1}	g = 1.904
	g = 1.91	J = 1.263 cm^{-1}
	g = 1.89	J = 1.647 cm^{-1}

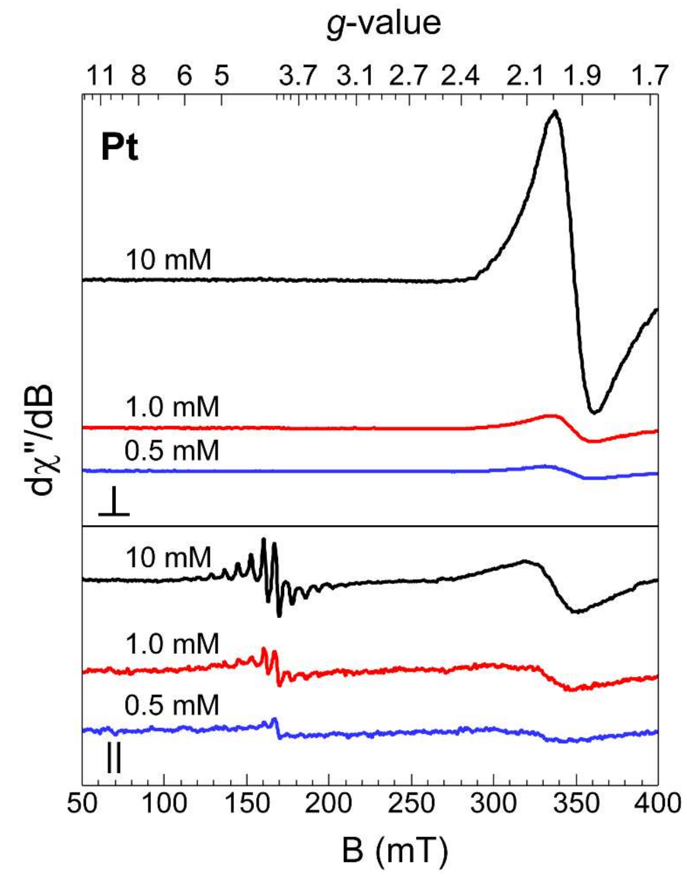

Figure S19. Perpendicular (⊥) and parallel (||) mode CW X-band EPR spectra for serial dilutions of **[VPtV](BArF$_{24}$)$_2$ (6)**. Parallel mode spectra are scaled 15x for ease of comparison to perpendicular mode data. Instrumental parameters: microwave frequency; perpendicular (⊥, 9.46 GHz), parallel (‖, 9.41 GHz); microwave power, 21 μW; modulation amplitude, 0.9 mT; Temperature, 4 K.

Table S2. Crystal Data and Structure Refinement for **[V'NiV'](BF$_4$)$_2$ (1), [VPdV](BF$_4$)$_2$ (2),** and **[VPtV](BF$_4$)$_2$ (3)**,

**M=**	**Ni**	**Pd**	**Pt**
Identification code	DJ_VNiVdacoNew2_autored	DJ_VPdV_I75_111922_0m_4a	DJVPtVI89_JAN1423_0ma
Empirical formula	$C_{22}H_{43}B_2F_8N_5NiO_2S_4V_2$	$C_{22}H_{42}B_2F_8N_6O_2PdS_4V_2$	$C_{22}H_{42}B_2F_8N_6O_2PtS_4V_2$
Formula weight	872.06	932.75	1021.44
Temperature (K)	100	120	110
Crystal system	Monoclinic	Monoclinic	Monoclinic
Space group	C2/c	$P2_1/c$	$P2_1/c$
a/Å	24.9516	14.2308	14.183
b/Å	10.9188	10.2867	10.2868
c/Å	12.4335	13.2241	13.234
α/°	90	90	90
β/°	99.094	116.702	116.397
γ/°	90	90	90
Volume/Å^3	3344.82	1729.4	1729.5
Z	4	2	2
ρ_{calc}, g/cm^3	1.732	1.791	1.961
μ/mm^{-1}	8.274	1.362	4.882
F(000)	1784	940	1004
Crystal size (mm^3)	0.3 x 0.3 x 0.1	0.3 x 0.3 x 0.3	0.3 x 0.3 x 0.3
Radiation	CuKα (λ=1.54184)	MoKα (λ=0.71073)	MoKα (λ=0.71073)
2Θ Range for Data Collection	3.588 to 80.058	2.624 to 25.391	2.548 to 25.418
Index ranges	-31≤h≤30, -13≤k≤12, -15≤l≤15	0≤h≤17, -12≤k≤12, -15≤l≤14	-17≤h≤17, -12≤k≤12, -15≤l≤15
Reflections collected	16776	6154	27027
Independent reflections	3570	3177	3174
Data/restraints/parameters	3570/0/212	3177/0/215	3174/0/215
Goodness-of-fit on F^2	1.129	1.114	1.026
Final R indexes (I ≥2σ(I)	R1=0.0452^a, wR2=0.1295^b	R1=0.0244^a, wR2 = 0.0561^b	R1=0.0158^a, wR2=0.0386^b
Final R indexes (all data)	R1=0.0461^a, wR2=0.1305^b	R1 = 0.0307^a, wR2= 0.0570^b	R1=0.0175^a, wR2=0.0396^b
Largest diffraction peak/hole, e Å^3	0.892/-0.863	0.917/-0.856	0.936/-0.450

^{a}R1=Σ(||F$_o$|-|F$_c$||)/Σ(F$_o$). ^{b}wR2=[Σ[w(F$_o^2$-F$_c^2$)^2]/[Σ(w(F$_o^2$)^2)]$^{1/2}$, w=1/[σ^2(F$_o^2$)+(ap)^2+bp], where p = (F$_o^2$+2F$_c^2$)/3.

Table S3. Crystal Data and Structure Refinement for **[V'NiV'](BArF$_{24}$)$_2$ (4), [VPdV](BArF$_{24}$)$_2$ (5)** and **[VPtV](BArF$_{24}$)$_2$ (6).**

**M=**	**Ni**	**Pd**	**Pt**
Identification code	KD_DJVNiVDacoBArF2	KD_DJ_VPdBArF_II94	DJ_VPtVBArF
Empirical formula	$C_{86}H_{68}B_2Cl_4F_{48}N_4NiO_2S_4V_2$	$C_{84}H_{64}B_2F_{48}Cl_4$ $N_4O_2PdS_4V_2$	$C_{84}H_{64}B_2Cl_4F_{48}N_4O_2PtS_4V_2$
Formula weight	2553.67	2573.33	2661.96
Temperature (K)	100	100.15	110
Crystal system	Monoclinic	Triclinic	Monoclinic
Space group	P-1	P-1	$P2_1/c$
a/Å	12.4949	10.3636	30.440
b/Å	13.3213	16.2862	9.7698
c/Å	16.8685	16.6376	36.189
α/°	95.150	116.95	90
β/°	103.949	103.59	113.117
γ/°	107.111	90.91	90
Volume/Å^3	2564.94	2408.8	9898
Z	1	1	4
ρ_{calc}, g/cm^3	1.691	1.751	1.775
μ/mm^{-1}	4.680	6.015	1.916
F(000)	1310	1261	5207
Crystal size (mm^3)	0.3 x 0.3 x 0.1	0.3 x 0.3 x 0.3	0.3 x 0.3 x 0.3
Radiation	CuKα (λ=1.54184)	CuKα (λ=1.54184)	MoKα (λ=0.71073)
2Θ Range for Data Collection	2.741 to 74.488	3.075 to 74.495	2.040 to 24.507
Index ranges	-15≤h≤15, -16≤k≤16, -21≤l≤19	-12≤h≤12, -13≤k≤20, -20≤l≤20	-35≤h≤35, -11<k<11, -42<l<41
Reflections collected	84121	42038	183552
Independent reflections	10455	3177	16403
Data/restraints/parameters	10455/587/796	9625/529/804	16403/865/1629
Goodness-of-fit on F^2	1.083	1.026	1.064
Final R indexes (I ≥2σ(I))	R1=0.0465^a, wR1=0.1229^b	R1 = 0.0422^a, wR1 = 0.1094^b	R1=0.0588^a, wR1=0.1235^b
Final R indexes (all data)	R1=0.0484^a, wR1=0.1246^b	R1 = 0.0441^a, wR1= 0.1112^b	R1=0.0942^a, wR1=0.1419^b
Largest diffraction peak/hole, e Å^3	0.844/-0.690	0.881/-0.757	1.269/-1.328

$^a R1=\Sigma(||F_o|-|F_c||)/\Sigma(F_o)$. $^b wR2=[\Sigma[w(F_o^2-F_c^2)^2]/[\Sigma(w(F_o^2)^2)]^{1/2}$, $w=1/[\sigma^2(F_o^2)+(ap)^2+bp]$, where $p = (F_o^2+2F_c^2)/3$.

Table S4. Experimental and computed metrical parameters for **[V'NiV']$^{2+}$**[a], **[VPdV]$^{2+}$**, and **[VPtV]$^{2+}$**.

	**[V'NiV']$^{2+}$**		**[VPdV]$^{2+}$**		**[VPtV]$^{2+}$**	
**Parameter**	**Exp.**	**Calc.**	**Exp.**	**Calc.**	**Exp.**	**Calc.**
**V-O distance (Å)**	1.593(19)	1.584	1.588	1.581	1.594	1.581
**V-V distance (Å)**	5.979(9)	6.197	6.272(11)	6.493	6.228(12)	6.430
**V-M distance (Å)**	2.990(4)	3.099	3.136(5)	3.246	3.114(6)	3.215
**V displacement (Å)**[b]	0.694	0.684	0.792	0.790	0.790	0.789
**τ_5 value (V)**	0.02	0.00	0.01	0.00	0.01	0.00
**τ_4 value (M)**	0.00	0.00	0.00	0.00	0.00	0.00
**Hinge (°)**[c]	97.9°	104.5°	102.3°	108.5°	100.6°	106.0°
**ΔE Singlet-Triplet (kcal/mol)**		0.6505		0.6526		0.6526
**J(cm^{-1}); (Calculated is NP)**[d]	0.282	0.010	0.954	0.242	1.372	0.513

Calculated coordinates were based on an earlier crystal structure with higher R value than the one used to extract the reported experimental coordinates.[a] Displacement of V atoms from the N_2S_2 best-fit plane.[b] Interplane angle between the MS_4 plane and the N_2S_2 best-fit plane.[c] Non-projected; formula is $(E_{LS} – E_{HS})/(2S_1S_2+S_2)$.[d]

Table S5. Selected Bond Lengths for **[V'NiV'](BF$_4$)$_2$ (1).**

Atom	Atom	Length/Å
V1	O1	1.593(19)
V1	N1	2.112(2)
V1	N2	2.112(2)
V1	S1	2.351(7)
V1	S2	2.352(7)
Ni00	S1	2.227(6)
Ni00	S2	2.228(6)

Table S6. Selected Bond Lengths for **[VPdV](BF$_4$)$_2$ (2).**

Atom	Atom	Length/Å
V1	O1	1.588(17)
V1	N1	2.088(18)
V1	N2	2.104(18)
V1	S1	2.370(8)
V1	S2	2.376(8)
Pd1	S1	2.330(6)
Pd1	S2	2.338(6)

Table S7. Selected Bond Lengths for **[VPtV](BF$_4$)$_2$. (3).**

Atom	Atom	Length/Å
V1	O1	1.594(18)
V1	N1	2.091(17)
V1	N2	2.107(17)
V1	S1	2.384(8)
V1	S2	2.375(7)
Pt1	S1	2.339(6)
Pt1	S2	2.331(6)

Table S8. Bond Angles for **[V'NiV'](BF$_4$)$_2$ (1).**

Atom1	Atom2	Atom3	Angle	Atom1	Atom2	Atom3	Angle
V1	Ni00	S1	51.05	S2	C10	C9	110.2(2)
V1	Ni00	S2	51.09	C3	C4	C5	117.7(2)
V1	Ni00	S1	128.95	S1	C1	C2	109.9(2)
V1	Ni00	S2	128.91	Ni00	V1	S1	47.45
S1	Ni00	S2	85.28	Ni00	V1	S2	47.47
S1	Ni00	V1	128.95	Ni00	V1	O1	144.04
S1	Ni00	S1	180	Ni00	V1	N1	99.57
S1	Ni00	S2	94.72	Ni00	V1	N2	98.88
S2	Ni00	V1	128.91	S1	V1	S2	79.83(2)
S2	Ni00	S1	94.72	S1	V1	O1	110.15(8)
S2	Ni00	S2	180	S1	V1	N1	86.10(6)
V1	Ni00	S1	51.05	S1	V1	N2	143.01(6)
V1	Ni00	S2	51.09	S2	V1	O1	108.95(8)
S1	Ni00	S2	85.28	S2	V1	N1	143.93(6)
Ni00	V1	S1	47.45	S2	V1	N2	86.13(6)
Ni00	V1	S2	47.47	O1	V1	N1	107.06(9)
Ni00	V1	O1	144.04	O1	V1	N2	106.76(9)
Ni00	V1	N1	99.57	N1	V1	N2	85.53(8)
Ni00	V1	N2	98.88	Ni00	S1	V1	81.5
S1	V1	S2	79.83(2)	Ni00	S1	C1	114.06
S1	V1	O1	110.15(8)	V1	S1	C1	98.41(8)
S1	V1	N1	86.10(6)	Ni00	S2	V1	81.44
S1	V1	N2	143.01(6)	Ni00	S2	C10	113.9
S2	V1	O1	108.95(8)	V1	S2	C10	98.51(8)
S2	V1	N1	143.93(6)	V1	N1	C3	106.3(2)
S2	V1	N2	86.13(6)	V1	N1	C6	116.2(1)
O1	V1	N1	107.06(9)	V1	N1	C2	106.8(1)
O1	V1	N2	106.76(9)	C3	N1	C6	109.5(2)
N1	V1	N2	85.53(8)	C3	N1	C2	108.1(2)
Ni00	S1	V1	81.5	C6	N1	C2	109.8(2)
Ni00	S1	C1	114.06	V1	N2	C8	115.7(1)
V1	S1	C1	98.41(8)	V1	N2	C9	107.1(1)
Ni00	S2	V1	81.44	V1	N2	C5	105.9(1)
Ni00	S2	C10	113.9	C8	N2	C9	110.2(2)
V1	S2	C10	98.51(8)	C8	N2	C5	109.5(2)
V1	N1	C3	106.3(2)	C9	N2	C5	108.2(2)
V1	N1	C6	116.2(1)	N2	C8	C7	113.4(2)
V1	N1	C2	106.8(1)	N1	C3	C4	113.2(2)
C3	N1	C6	109.5(2)	C8	C7	C6	117.6(2)

C3	N1	C2	108.1(2)	N1	C6	C7	113.2(2)
C6	N1	C2	109.8(2)	N1	C2	C1	111.6(2)
V1	N2	C8	115.7(1)	N2	C9	C10	112.7(2)
V1	N2	C9	107.1(1)	N2	C5	C4	113.9(2)
V1	N2	C5	105.9(1)	S2	C10	C9	110.2(2)
C8	N2	C9	110.2(2)	C3	C4	C5	117.7(2)
C8	N2	C5	109.5(2)	S1	C1	C2	109.9(2)
C9	N2	C5	108.2(2)	F4	B1	F3	110.1(3)
N2	C8	C7	113.4(2)	F4	B1	F1	107.6(3)
N1	C3	C4	113.2(2)	F4	B1	F2	110.0(3)
C8	C7	C6	117.6(2)	F3	B1	F1	107.9(3)
N1	C6	C7	113.2(2)	F3	B1	F2	110.4(3)
N1	C2	C1	111.6(2)	F1	B1	F2	110.9(3)
N2	C9	C10	112.7(2)	N00B	C00L	C00P	180
N2	C5	C4	113.9(2)				

Table S9. Bond Angles for **[VPdV](BF_4)$_2$ (2).**

Atom1	Atom2	Atom3	Angle	Atom1	Atom2	Atom3	Angle
V1	Pd1	S1	48.69	N2	C5	C4	113.2(2)
V1	Pd1	S2	48.83	C5	C4	C3	115.1(2)
V1	Pd1	S1	131.31	N1	C3	C4	113.1(2)
V1	Pd1	S2	131.17	Pd1	V1	S1	47.61
S1	Pd1	S2	85.72	Pd1	V1	S2	47.77
S1	Pd1	V1	131.31	Pd1	V1	O1	144.27
S1	Pd1	S1	180	Pd1	V1	N1	97.62
S1	Pd1	S2	94.28	Pd1	V1	N2	96.97
S2	Pd1	V1	131.17	S1	V1	S2	83.98(2)
S2	Pd1	S1	94.28	S1	V1	O1	109.98(7)
S2	Pd1	S2	180	S1	V1	N1	85.23(6)
V1	Pd1	S1	48.69	S1	V1	N2	137.81(6)
V1	Pd1	S2	48.83	S2	V1	O1	112.56(7)
S1	Pd1	S2	85.72	S2	V1	N1	138.53(6)
Pd1	V1	S1	47.61	S2	V1	N2	84.77(6)
Pd1	V1	S2	47.77	O1	V1	N1	108.77(9)
Pd1	V1	O1	144.27	O1	V1	N2	111.90(9)
Pd1	V1	N1	97.62	N1	V1	N2	76.94(8)
Pd1	V1	N2	96.97	Pd1	S1	V1	83.7
S1	V1	S2	83.98(2)	Pd1	S1	C1	109.03
S1	V1	O1	109.98(7)	V1	S1	C1	99.38(8)
S1	V1	N1	85.23(6)	Pd1	S2	V1	83.4

S1	V1	N2	137.81(6)	Pd1	S2	C9	108.58
S2	V1	O1	112.56(7)	V1	S2	C9	99.67(8)
S2	V1	N1	138.53(6)	V1	N1	C2	111.3(1)
S2	V1	N2	84.77(6)	V1	N1	C7	104.8(1)
O1	V1	N1	108.77(9)	V1	N1	C3	110.9(1)
O1	V1	N2	111.90(9)	C2	N1	C7	112.2(2)
N1	V1	N2	76.94(8)	C2	N1	C3	107.3(2)
Pd1	S1	V1	83.7	C7	N1	C3	110.4(2)
Pd1	S1	C1	109.03	V1	N2	C8	112.3(1)
V1	S1	C1	99.38(8)	V1	N2	C6	104.2(1)
Pd1	S2	V1	83.4	V1	N2	C5	110.7(2)
Pd1	S2	C9	108.58	C8	N2	C6	112.3(2)
V1	S2	C9	99.67(8)	C8	N2	C5	107.2(2)
V1	N1	C2	111.3(1)	C6	N2	C5	110.2(2)
V1	N1	C7	104.8(1)	S2	C9	C8	111.6(2)
V1	N1	C3	110.9(1)	N2	C8	C9	113.1(2)
C2	N1	C7	112.2(2)	N1	C2	C1	113.3(2)
C2	N1	C3	107.3(2)	N1	C7	C6	110.5(2)
C7	N1	C3	110.4(2)	S1	C1	C2	110.5(2)
V1	N2	C8	112.3(1)	N2	C6	C7	110.9(2)
V1	N2	C6	104.2(1)	N2	C5	C4	113.2(2)
V1	N2	C5	110.7(2)	C5	C4	C3	115.1(2)
C8	N2	C6	112.3(2)	N1	C3	C4	113.1(2)
C8	N2	C5	107.2(2)	F3	B1	F4	110.2(2)
C6	N2	C5	110.2(2)	F3	B1	F2	109.5(2)
S2	C9	C8	111.6(2)	F3	B1	F1	109.4(2)
N2	C8	C9	113.1(2)	F4	B1	F2	111.3(2)
N1	C2	C1	113.3(2)	F4	B1	F1	108.5(2)
N1	C7	C6	110.5(2)	F2	B1	F1	108.0(2)
S1	C1	C2	110.5(2)	N3	C10	C11	179.6(3)
N2	C6	C7	110.9(2)				

Table S10. Bond Angles for **[VPtV](BF_4)$_2$ (3).**

Atom1	Atom2	Atom3	Angle	Atom1	Atom2	Atom3	Angle
V1	Pt1	S1	49.39	N2	C6	C7	110.7(2)
V1	Pt1	S2	49.17	N2	C5	C4	112.9(2)
V1	Pt1	S1	130.61	N1	C3	C4	112.9(2)
V1	Pt1	S2	130.83	Pt1	V1	S1	48.13
S1	Pt1	S2	85.95	Pt1	V1	S2	47.97
S1	Pt1	V1	130.61	Pt1	V1	O1	144.89
S1	Pt1	S1	180	Pt1	V1	N2	96.56
S1	Pt1	S2	94.05	Pt1	V1	N1	97.24
S2	Pt1	V1	130.83	S1	V1	S2	83.96(2)
S2	Pt1	S1	94.05	S1	V1	O1	112.48(6)
S2	Pt1	S2	180	S1	V1	N2	84.99(5)
V1	Pt1	S1	49.39	S1	V1	N1	138.74(6)
V1	Pt1	S2	49.17	S2	V1	O1	109.76(6)
S1	Pt1	S2	85.95	S2	V1	N2	138.00(6)
Pt1	V1	S1	48.13	S2	V1	N1	85.49(6)
Pt1	V1	S2	47.97	O1	V1	N2	111.92(8)
Pt1	V1	O1	144.89	O1	V1	N1	108.64(8)
Pt1	V1	N2	96.56	N2	V1	N1	76.75(7)
Pt1	V1	N1	97.24	Pt1	S1	V1	82.47
S1	V1	S2	83.96(2)	Pt1	S1	C9	107.87
S1	V1	O1	112.48(6)	V1	S1	C9	99.39(8)
S1	V1	N2	84.99(5)	Pt1	S2	V1	82.86
S1	V1	N1	138.74(6)	Pt1	S2	C1	108.33
S2	V1	O1	109.76(6)	V1	S2	C1	98.97(8)
S2	V1	N2	138.00(6)	V1	N2	C8	112.0(1)
S2	V1	N1	85.49(6)	V1	N2	C6	104.6(1)
O1	V1	N2	111.92(8)	V1	N2	C5	110.5(1)
O1	V1	N1	108.64(8)	C8	N2	C6	112.4(2)
N2	V1	N1	76.75(7)	C8	N2	C5	106.9(2)
Pt1	S1	V1	82.47	C6	N2	C5	110.5(2)
Pt1	S1	C9	107.87	V1	N1	C7	105.0(1)
V1	S1	C9	99.39(8)	V1	N1	C2	110.9(1)
Pt1	S2	V1	82.86	V1	N1	C3	110.6(1)
Pt1	S2	C1	108.33	C7	N1	C2	112.7(2)
V1	S2	C1	98.97(8)	C7	N1	C3	110.7(2)
V1	N2	C8	112.0(1)	C2	N1	C3	107.0(2)
V1	N2	C6	104.6(1)	S1	C9	C8	111.5(2)
V1	N2	C5	110.5(1)	N2	C8	C9	113.5(2)
C8	N2	C6	112.4(2)	S2	C1	C2	110.6(2)

C8	N2	C5	106.9(2)	N1	C7	C6	110.5(2)
C6	N2	C5	110.5(2)	N1	C2	C1	113.4(2)
V1	N1	C7	105.0(1)	C5	C4	C3	115.0(2)
V1	N1	C2	110.9(1)	N2	C6	C7	110.7(2)
V1	N1	C3	110.6(1)	N2	C5	C4	112.9(2)
C7	N1	C2	112.7(2)	N1	C3	C4	112.9(2)
C7	N1	C3	110.7(2)	F3	B1	F1	110.3(2)
C2	N1	C3	107.0(2)	F3	B1	F2	111.4(2)
S1	C9	C8	111.5(2)	F3	B1	F4	108.9(2)
N2	C8	C9	113.5(2)	F1	B1	F2	108.9(2)
S2	C1	C2	110.6(2)	F1	B1	F4	109.2(2)
N1	C7	C6	110.5(2)	F2	B1	F4	107.9(2)
N1	C2	C1	113.4(2)	N3	C10	C11	179.5(3)
C5	C4	C3	115.0(2)				

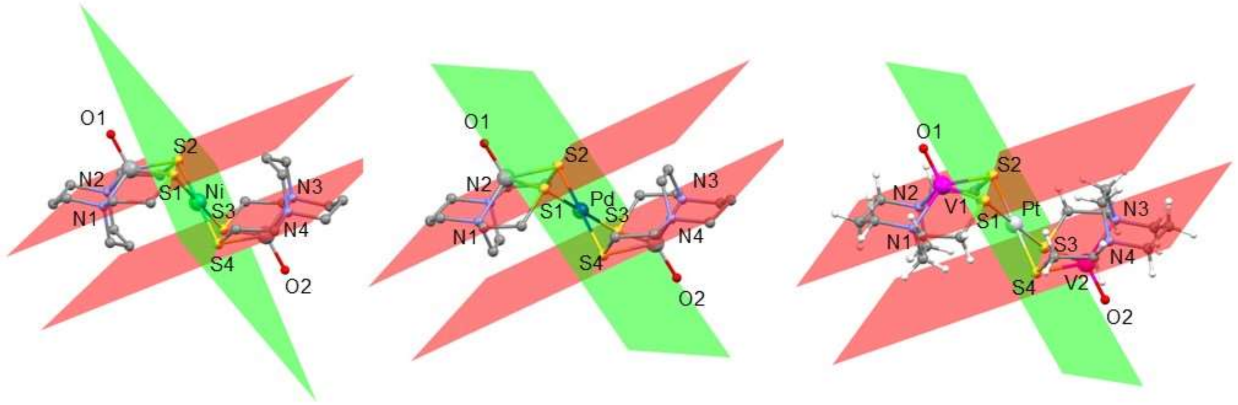

Figure S20. N_2S_2 (red) and MS_4 (green) best planes calculated through Mercury for **1**, **2** and **3**.

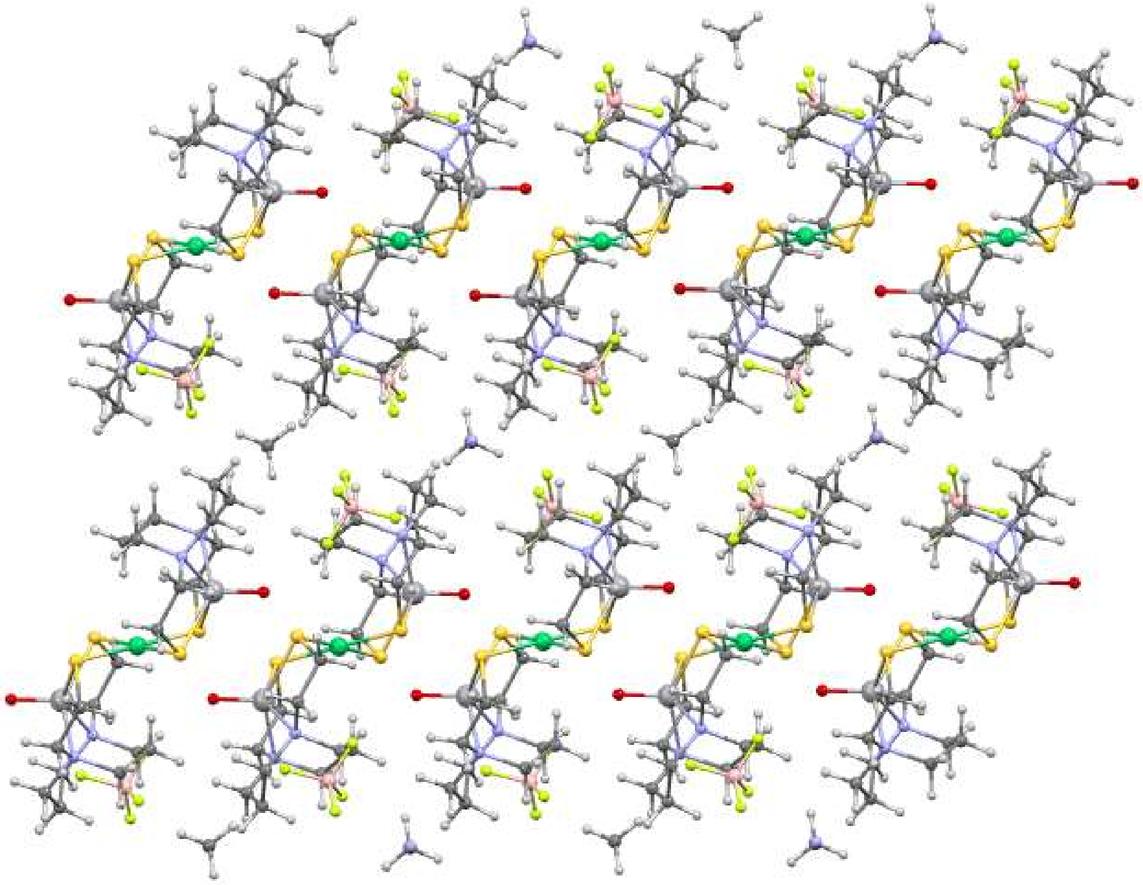

Figure S21. Crystal packing diagram of **[V′NiV′](BF$_4$)$_2$ (1)** looking down the *b* axis.

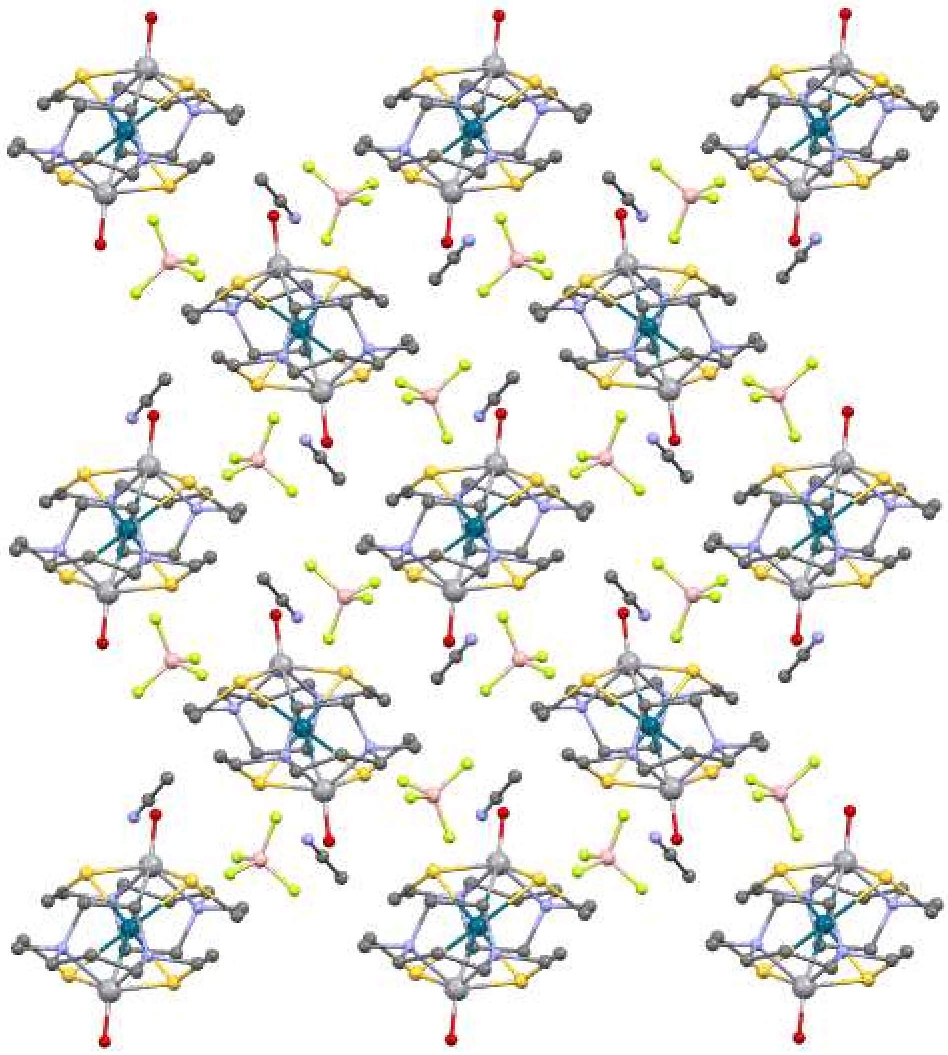

Figure S22. Crystal packing diagram of **[VPdV](BF$_4$)$_2$ (2)** looking down the *a* axis.

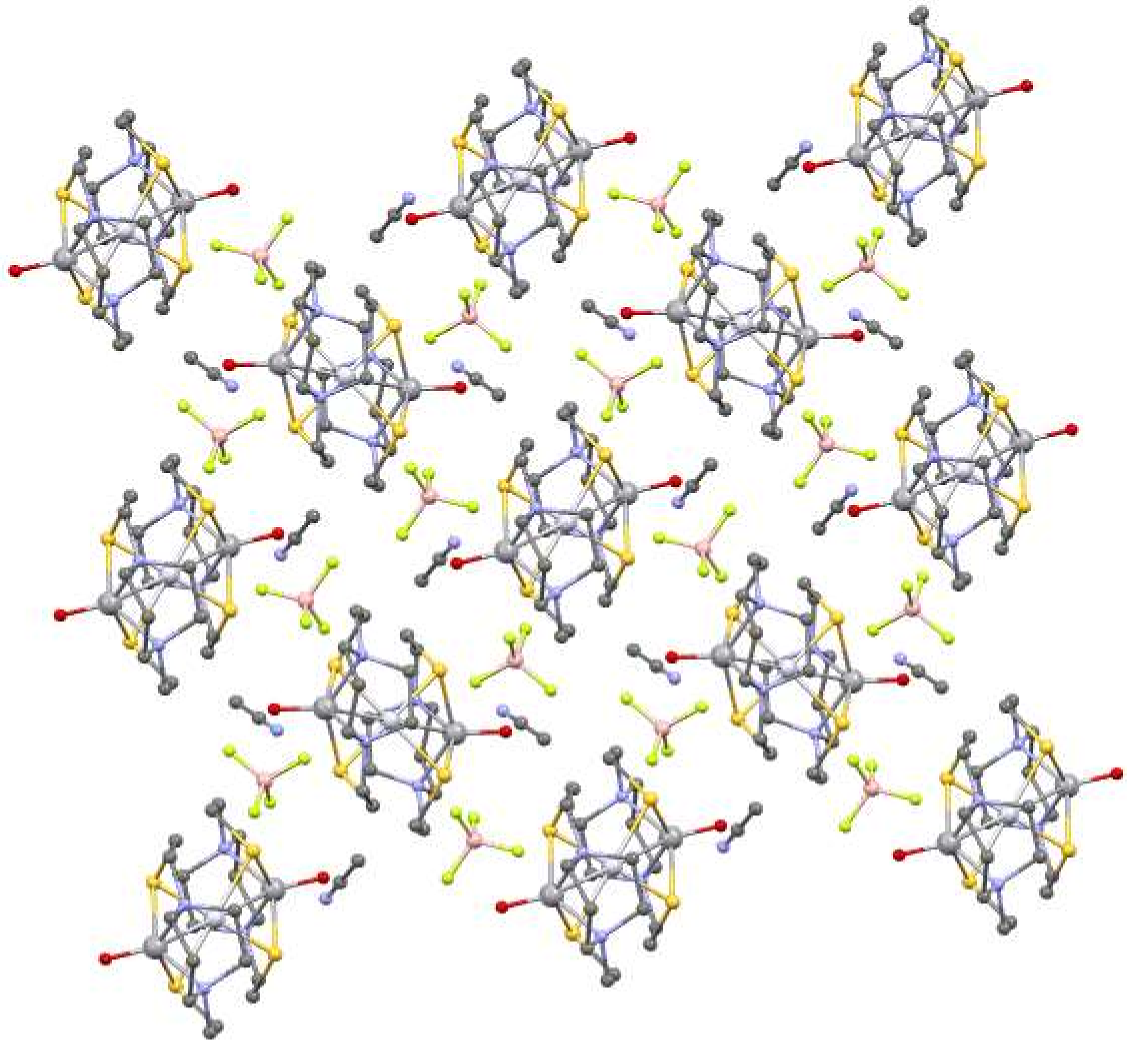

Figure S23. Crystal packing diagram of **[VPtV](BF$_4$)$_2$ (3)** looking down the *a* axis.

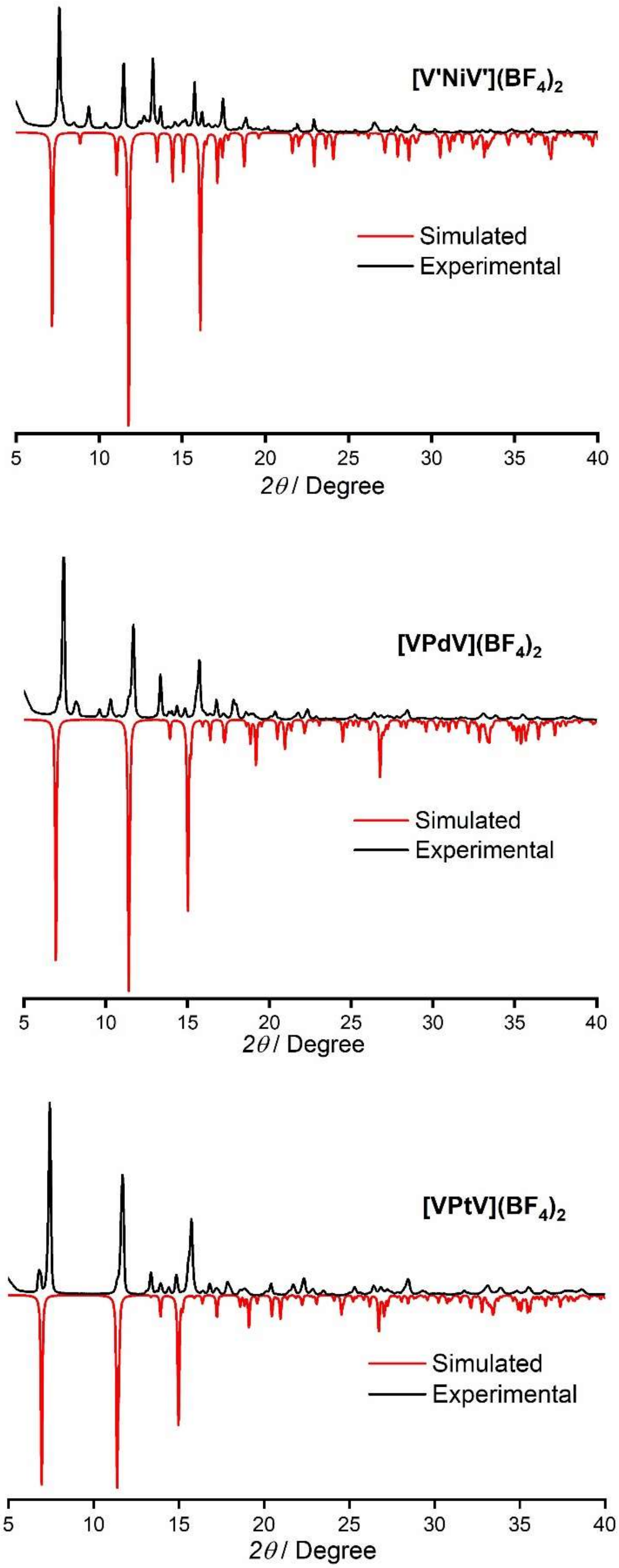

Figure S24. Powder X-ray diffraction patterns of **1, 2** and **3** compared to Mercury simulations.

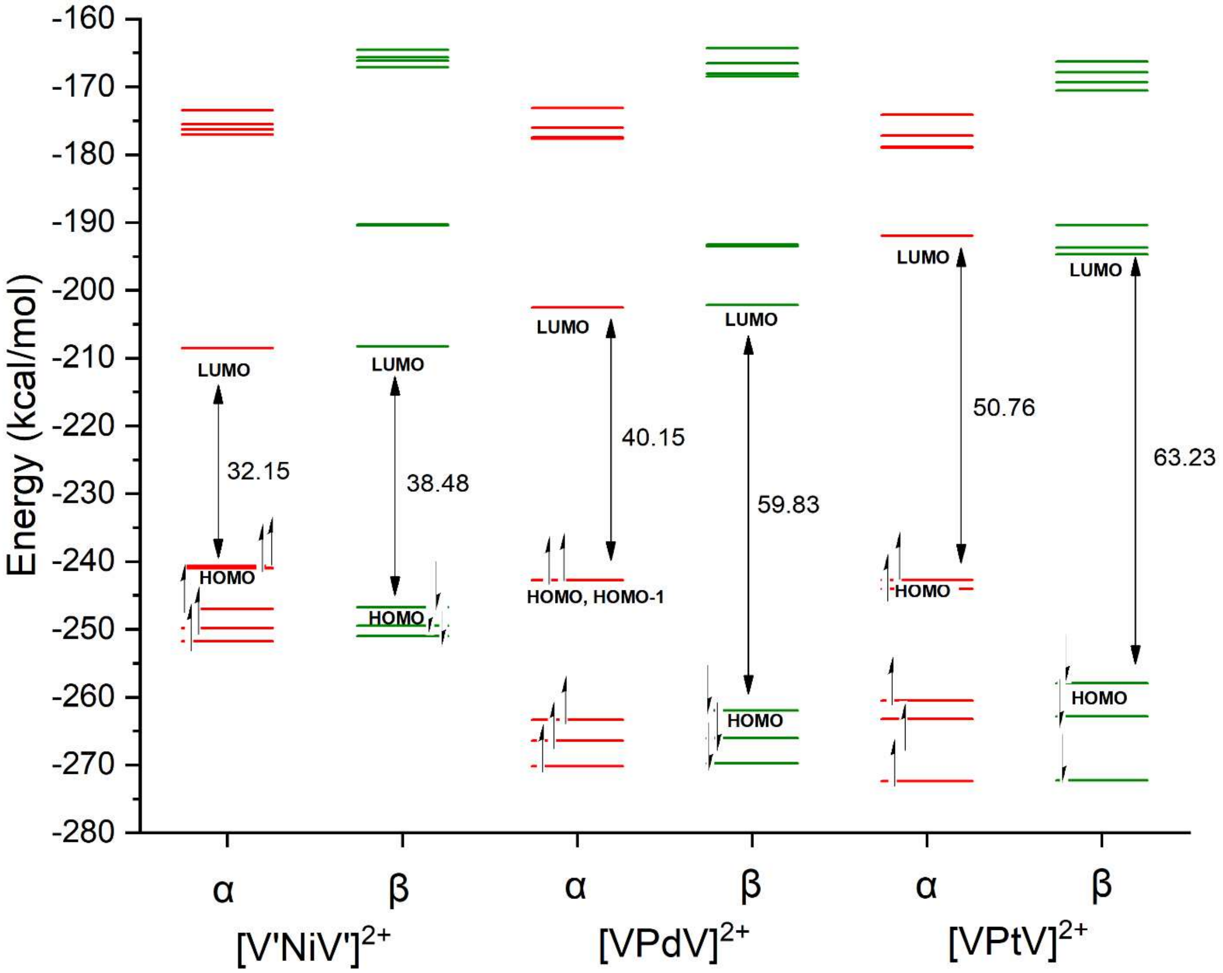

Figure S25. Alpha and Beta MO energy levels from HOMO-4 to LUMO+4 for the triplet (ground) state of the cations in the **[VMV]$^{2+}$** series.

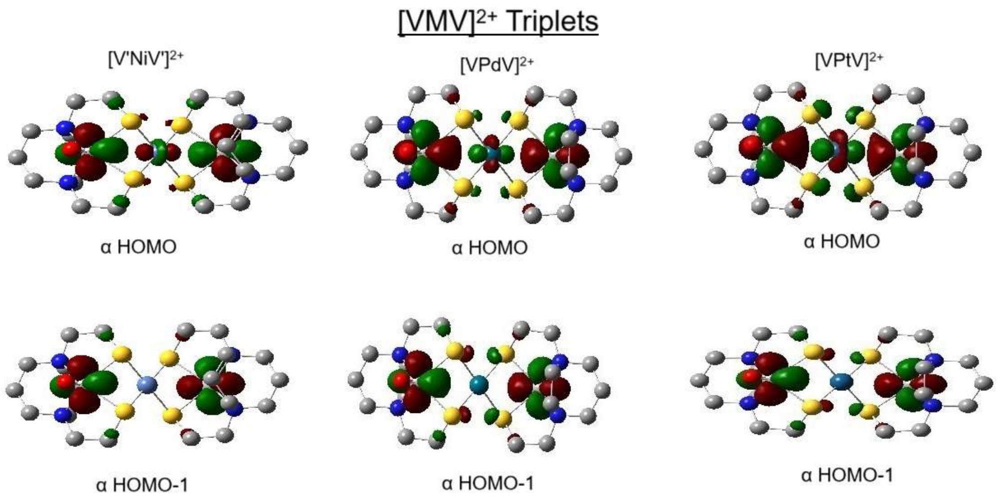

Figure S26. Alpha HOMO and HOMO-1 surface density plots (isovalue = 0.03) for the **[VMV]$^{2+}$** cation series.

Computational Coordinates (Optimized triplet) [V′NiV′]$^{2+}$ (1)

	X	Y	Z		X	Y	Z
Ni	-6E-07	-1E-07	9E-07	V	2.933989	-8.1E-06	-0.99642
V	-2.93399	0.000008	0.996424	S	1.129748	1.537431	-1.20331
S	-1.12975	-1.53743	1.203309	S	1.129661	-1.53747	-1.20344
S	-1.12966	1.537469	1.203444	O	3.80301	0.000431	-2.32091
O	-3.80301	-0.00043	2.320912	N	3.858597	-1.45009	0.289609
N	-3.8586	1.45009	-0.28961	N	3.858751	1.449927	0.289657
N	-3.85875	-1.44993	-0.28966	C	1.876151	-2.92602	-0.19435
C	-1.87615	2.92602	0.194352	H	1.581909	-3.86435	-0.67329
H	-1.58191	3.864348	0.673289	H	1.44582	-2.89397	0.809695
H	-1.44582	2.893971	-0.80969	C	1.876404	2.925945	-0.19428
C	-1.87641	-2.92594	0.194279	H	1.446174	2.893914	0.809812
H	-1.44617	-2.89391	-0.80981	H	1.58217	3.864291	-0.67318
H	-1.58217	-3.86429	0.673179	C	3.556103	1.299688	1.771665
C	-3.5561	-1.29969	-1.77167	H	4.495817	1.379968	2.329091
H	-4.49581	-1.37997	-2.32909	H	2.933552	2.144649	2.077728
H	-2.93355	-2.14465	-2.07773	C	3.399679	2.801206	-0.20444
C	-3.39968	-2.80121	0.204443	H	3.846664	3.590278	0.419174
H	-3.84666	-3.59028	-0.41917	H	3.778074	2.914661	-1.22556
H	-3.77807	-2.91466	1.225559	C	2.818347	4.37E-05	2.1034
C	-2.81835	-4.4E-05	-2.1034	H	1.840397	7.65E-05	1.582588
H	-1.8404	-7.5E-05	-1.58259	H	2.588625	-3.6E-05	3.176568
H	-2.58862	3.62E-05	-3.17657	C	3.399448	-2.80137	-0.20434
C	-3.39945	2.80137	0.204344	H	3.777925	-2.91501	-1.22541
H	-3.77792	2.915007	1.22541	H	3.846293	-3.59045	0.419382
H	-3.84629	3.590451	-0.41938	C	5.352427	-1.31464	0.037276
C	-5.35243	1.314637	-0.03727	H	5.857787	-2.15453	0.53501
H	-5.85779	2.154531	-0.53501	H	5.497969	-1.41028	-1.04211
H	-5.49797	1.410274	1.042116	C	3.556089	-1.29962	1.771602
C	-3.55609	1.29962	-1.7716	H	4.495854	-1.37987	2.328984
H	-4.49586	1.379871	-2.32898	H	2.933538	-2.14454	2.077819
H	-2.93354	2.144541	-2.07782	C	5.352624	1.314388	0.037509
C	-5.35262	-1.31439	-0.03751	H	5.498356	1.410255	-1.04183
H	-5.49836	-1.41026	1.041832	H	5.857994	2.154117	0.535511
H	-5.85799	-2.15412	-0.53551	C	5.953662	-0.00022	0.557505
C	-5.95366	0.000225	-0.55751	H	5.967714	-0.00036	1.653794
H	-5.96771	0.000365	-1.65379	H	7.009073	-0.00028	0.257813
H	-7.00907	0.000283	-0.25781				

Computational Coordinates (Optimized triplet)
$[VPdV]^{2+}$ (2)

	X	Y	Z		X	Y	Z
Pd	-3.7E-06	-0.0001	1E-07	V	3.076136	-7.8E-06	1.037089
V	-3.07614	-5.6E-05	-1.03709	S	1.306667	-1.61676	1.150041
S	-1.30653	1.616544	-1.1502	S	1.306556	1.616598	1.150091
S	-1.30668	-1.61679	-1.14995	O	3.958464	5.56E-05	2.349329
O	-3.95848	-0.00024	-2.34932	N	3.955096	-1.31765	-0.38582
N	-3.955	1.317802	0.385695	N	3.955036	1.317756	-0.38575
N	-3.95512	-1.3176	0.385891	C	1.976309	2.865141	-0.07034
C	-1.97653	-2.86518	0.070603	H	1.723741	3.856661	0.316153
H	-1.72403	-3.85675	-0.31582	H	1.4612	2.725239	-1.02323
H	-1.46138	-2.72522	1.023488	C	3.492868	2.737329	-0.17447
C	-3.49307	-2.73723	0.174744	H	3.959616	3.06962	0.75827
H	-3.95985	-3.06956	-0.75797	H	3.871642	3.370458	-0.99071
H	-3.87188	-3.37024	0.991049	C	3.493014	-2.73727	-0.17465
C	-3.49281	2.737357	0.174365	H	3.871811	-3.3703	-0.99096
H	-3.87157	3.370506	0.99058	H	3.959816	-3.06961	0.758063
H	-3.95957	3.069618	-0.7584	C	3.542123	-0.77929	-1.73048
C	-3.54207	0.779549	1.730407	H	4.200849	-1.1845	-2.50814
H	-4.20074	1.184876	2.508034	H	2.527962	-1.13483	-1.92861
H	-2.52787	1.135039	1.928493	C	1.976477	-2.86518	-0.07048
C	-1.97626	2.865141	0.070198	H	1.461328	-2.72524	-1.02336
H	-1.46114	2.72526	1.023094	H	1.723977	-3.85674	0.315961
H	-1.72367	3.856644	-0.31634	C	3.542089	0.779453	-1.73044
C	-3.54213	-0.77919	1.730526	H	2.527897	1.134951	-1.92854
H	-2.52798	-1.13475	1.92866	H	4.200776	1.184734	-2.50809
H	-4.20085	-1.18434	2.508211	C	5.468404	1.292151	-0.23149
C	-5.46849	-1.29191	0.231644	H	5.675249	1.433965	0.833036
H	-5.67537	-1.43381	-0.83288	H	5.879794	2.146378	-0.78634
H	-5.87992	-2.14606	0.786578	C	6.092639	0.000118	-0.76193
C	-6.09264	0.000227	0.761953	H	6.081207	0.000144	-1.85864
H	-6.08121	0.000313	1.858661	H	7.151333	0.000143	-0.47762
H	-7.15134	0.00026	0.477646	C	5.468467	-1.29199	-0.23156
C	-5.46837	1.292223	0.231442	H	5.675321	-1.43382	0.832976
H	-5.67523	1.433982	-0.83309	H	5.879892	-2.14616	-0.78644
H	-5.87973	2.146483	0.786241				

Computational Coordinates (Optimized triplet)
$[VPtV]^{2+}$ (3)

	X	Y	Z		X	Y	Z
Pt	-7.3E-06	7.38E-05	9.65E-05	V	3.040313	2.07E-05	1.04516
V	-3.04025	4.4E-06	-1.0452	S	1.267368	-1.61726	1.186115
S	-1.26739	1.617403	-1.18591	S	1.267474	1.617418	1.185977
S	-1.26729	-1.61727	-1.18599	O	3.946122	0.000173	2.340999
O	-3.94596	-0.00012	-2.34111	N	3.90053	-1.31718	-0.38747
N	-3.90065	1.317059	0.387449	N	3.900606	1.317034	-0.38761
N	-3.90056	-1.31716	0.387423	C	1.9318	-2.87135	-0.03603
C	-1.932	2.871379	0.03626	H	1.400999	-2.73755	-0.98098
H	-1.40126	2.737549	0.981247	H	1.690445	-3.86103	0.36204
H	-1.69068	3.8611	-0.36173	C	3.445663	-2.73705	-0.16458
C	-3.44586	2.736971	0.164686	H	3.813059	-3.37182	-0.98478
H	-3.81336	3.371666	0.984902	H	3.929115	-3.06538	0.761087
H	-3.92926	3.065333	-0.76099	C	1.931966	2.871364	-0.03629
C	-1.9318	-2.87134	0.036149	H	1.690668	3.861107	0.361653
H	-1.69041	-3.86104	-0.36184	H	1.401164	2.737485	-0.98123
H	-1.40107	-2.73749	0.98114	C	3.462694	0.779326	-1.72488
C	-3.46279	-0.77951	1.724753	H	2.443196	1.131322	-1.90096
H	-2.44328	-1.13145	1.900892	H	4.106013	1.185717	-2.51471
H	-4.10614	-1.18599	2.514509	C	3.445813	2.736941	-0.16478
C	-3.44566	-2.73702	0.164536	H	3.929246	3.065241	0.760908
H	-3.92901	-3.06529	-0.76121	H	3.813306	3.37167	-0.98497
H	-3.81318	-3.37183	0.984654	C	6.031177	-0.00015	-0.80234
C	-6.03125	-0.00014	0.802076	H	7.094943	-0.00016	-0.53766
H	-7.09499	-0.00017	0.537313	H	5.999028	-0.00021	-1.8986
H	-5.99918	-0.00014	1.898333	C	3.462705	-0.77959	-1.72481
C	-3.4629	0.77941	1.724786	H	4.106096	-1.18605	-2.51454
H	-4.10637	1.185779	2.514501	H	2.443227	-1.13162	-1.90097
H	-2.44345	1.131486	1.901045	C	5.416422	-1.29119	-0.25967
C	-5.41654	1.290978	0.25954	H	5.641986	-1.43166	0.80125
H	-5.64203	1.431506	-0.80139	H	5.817702	-2.14627	-0.82054
H	-5.81791	2.145993	0.820429	C	5.41649	1.290989	-0.25984
C	-5.41644	-1.2912	0.259533	H	5.817812	2.145971	-0.82081
H	-5.81775	-2.14624	0.820424	H	5.642069	1.431592	0.801068
